# Porcine Epidemic Diarrhea Virus and the Host Innate Immune Response

**DOI:** 10.3390/pathogens9050367

**Published:** 2020-05-11

**Authors:** Shasha Li, Jinping Yang, Zixiang Zhu, Haixue Zheng

**Affiliations:** State Key Laboratory of Veterinary Etiological Biology, National Foot and Mouth Diseases Reference Laboratory, Key Laboratory of Animal Virology of Ministry of Agriculture, Lanzhou Veterinary Research Institute, Chinese Academy of Agricultural Sciences, Lanzhou 730046, China; lishsh91@163.com (S.L.); Yangjinpingyjs@163.com (J.Y.)

**Keywords:** coronavirus, porcine epidemic diarrhea virus, innate immunity, type I IFN, immune evasion

## Abstract

Porcine epidemic diarrhea virus (PEDV), a swine enteropathogenic coronavirus (CoV), is the causative agent of porcine epidemic diarrhea (PED). PED causes lethal watery diarrhea in piglets, which has led to substantial economic losses in many countries and is a great threat to the global swine industry. Interferons (IFNs) are major cytokines involved in host innate immune defense, which induce the expression of a broad range of antiviral effectors that help host to control and antagonize viral infections. PEDV infection does not elicit a robust IFN response, and some of the mechanisms used by the virus to counteract the host innate immune response have been unraveled. PEDV evades the host innate immune response by two main strategies including: 1) encoding IFN antagonists to disrupt innate immune pathway, and 2) hiding its viral RNA to avoid the exposure of viral RNA to immune sensors. This review highlights the immune evasion mechanisms employed by PEDV, which provides insights for the better understanding of PEDV-host interactions and developing effective vaccines and antivirals against CoVs.

## 1. Introduction

Porcine epidemic diarrhea virus (PEDV) is the etiological agent of porcine epidemic diarrhea (PED) that causes an acute and highly contagious enteric disease of swine characterized by vomiting, diarrhea, dehydration, and anorexia in pigs of all ages, especially resulting in severe diarrhea and high mortality rate in piglets. In serious cases, outbreak of PED even leads to a mortality rate of 100% in pigs [1,2,3]. The causative agent of PED was first described in the 1970s in England [4]. In 1976, an unrecognized enteric disease was reported in several European countries [5]. The viral diarrhea was collectively designated “PED” in 1982 [6]. Endemic PED had been described in both developed and developing countries, but with a low impact on the swine industry until 2010. In 2010, outbreaks of PED caused by highly pathogenic variant PEDV strains occurred in China, and this was immediately reported in other Asian counties, causing up to 100% mortality in suckling piglets [1,7,8,9]. In April 2013, PEDV entered the United States (US) for the first time and the virulent strains spread rapidly across the US [10]. Apart from almost 100% mortality rate in suckling piglets, PEDV infection also damaged the growth performance of finishing pigs [11]. The highly pathogenic strains of PEDV spread worldwide, resulting in serious problems to the swine industry and substantial economic losses [7,10,12,13]. Vaccination used to be the main strategy to prevent and control the rate of PEDV infection [14], however, the current available PEDV vaccines cannot provide complete protection for the pigs affected by the highly pathogenic strains. The optimal vaccines should induce efficient maternal antibodies in sows that could be transmitted to the offspring and protect neonatal suckling piglets from PEDV. The vaccination method is critically associated with the antibodies’ induction by PEDV vaccines. Oral vaccination with attenuated PEDV seems to be more efficacious than injectable vaccine. Oral immunization of PEDV-seronegative pregnant sows with attenuated PEDV leads to lower mortality rate of piglets and higher amounts of IgA transferred from the sows to the offspring than that in the intramuscularly injected group. However, the duration of virus shedding after challenge was not reduced compared to the control group [15]. The immunization dose of the virus for sows and the challenge dose of the virus for piglets significantly affected the protection against virus challenge [16]. Moreover, epidemiological studies have demonstrated that the prevalent strains causing previous PED outbreaks in China in 2010 and the recent outbreaks in North America and Asia all belong to the genogroup II (GII) PEDV and the S gene of GIIb PEDV contains typical insertion and deletion mutations [2]. Therefore, new PEDV vaccines should be developed based on these variant strains. The biological characteristics, pathogenicity and immune protective effects of GIIb PEDV strains should be studied as soon as possible. An inactivated vaccine based on genotype IIb strain AJ1102 developed by Wuhan Keqian company was approved for clinical trial in 2014. In addition, the lactic acid bacteria (LAB) that have the ability to interact with the intestinal cells can be used as a potential vector for improving delivery of oral vaccines [17]. Developing the transgenic plants expressing antigenic proteins as edible vaccines to induce efficient mucosal immune responses might be a prominent strategy to prevent PEDV infection [18]. Choosing appropriate and effective adjuvants for PEDV vaccines to enhance adaptive immune response is also important. In addition, the construction of recombinant PEDV with deletion or mutation of the immune antagonistic regions or sites is a potential strategy to develop PEDV vaccines as well. The produced modified antigens will induce an increased and earlier immune response in pigs, compared with the antigens from wildtype viruses. Therefore, the modification of viral antigens, the optimization of immunization methods, screening effective adjuvants, and exploiting a new generation of vaccines are promising directions for the control of PED.PEDV is classified as a coronavirus (CoV), due to its similar electron microscopic appearance and polypeptide structure to other CoVs [19,20,21]. CoVs (subfamily *Coronavirinae*, family *Coronaviridae*, order *Nidovirales*) are a large family of viruses that have broad host ranges and have caused global public health issues [22]. The currently known CoVs are divided into four genera, which include *Alphacoronavirus* (*α*-CoV), *Betacoronavirus* (*β*-CoV), *Gammacoronavirus* (*γ*-CoV) and *Deltacoronavirus* (*δ*-CoV), according to the genome phylogeny and serotypes. CoVs give rise to a wide spectrum of diseases in humans and animals, but generally fall into two classes, with respiratory or enteric tropisms. Moreover, the *β*-CoV genus consists of a lot of important CoVs that result in serious respiratory illnesses in humans, such as severe acute respiratory syndrome coronavirus (SARS-CoV), Middle East respiratory syndrome coronavirus (MERS-CoV), and the latest 2019 novel coronavirus (SARS-CoV2). SARS-CoV resulted in an outbreak of severe respiratory disease through China in 2002–2003 [23,24]. MERS-CoV was first identified in Saudi Arabia in 2012 and is associated with severe pneumonia and acute respiratory distress syndrome (ARDS) that results in a high mortality (~35%) [25,26]. SARS-CoV2 was identified in Wuhan, China in December 2019, which leads to similar symptoms in humans as SARS-CoV, but it is more infectious to humans than SARS-CoV [27,28]. Furthermore, *α*-CoV genus includes porcine respiratory coronavirus (PRCV) and various enteric CoVs that lead to viral diarrhea in pigs, such as PEDV, transmissible gastroenteritis virus (TGEV), and HKU2-like porcine enteric alphacoronavirus (PEAV) [29,30]. PRCV infects the upper respiratory epithelial cells and alveolar macrophages, but the clinical manifestations caused by PRCV are not significant [31]. The swine enteric CoVs cause severe diarrhea, vomiting, and mortality in piglets, having contributed to enormous economic losses to the global swine industry [6].

PEDV is the main causative pathogen of viral diarrhea in piglets. Over the years, significant work has been done to investigate PEDV pathogenesis and prevention. Aminopeptidase N protein (APN, also known as CD13) is identified as a functional receptor of PEDV and TGEV [32,33,34]. APN, a 150 KD type II transmembrane glycoprotein, is mainly expressed on the apical membrane of mature enterocytes [35]. APN binds to the 477-629 amino-acid region in the C-terminal region of PEDV spike 1 (S1) protein [34]. Apart from APN, PEDV is able to bind to sialic acids [36]. It remains unknown whether PEDV uses sugar coreceptors during viral infection [34,36]. PEDV infects multiple cell lines from different species including bat and primate (human and non-human) in vitro. The ability of PEDV to infect the cells of different species indicates that the virus utilizes the evolutionarily conserved cell components as receptors, thereby enhancing the potential for cross-species and potentially, zoonotic transmission [37,38]. The highly pathogenic variant strains of PEDV were identified in 2010 and caused a high morbidity of up to 100% in piglets, and since then, these strains become dominant, leading to most of the acute outbreaks of PED worldwide [1,7,8]. The high virulence of these strains is critically associated with the immune evasion mechanisms employed by the virus. PEDV has evolved different strategies to delicately manipulate and damage the host innate immune system for their multiplication. Clarification of these mechanisms is critical for understanding the host range, tropisms, pathogenesis, and for developing effective vaccines and antiviral drugs to curb the spread of PEDV in pigs. In this review, we provide an overview of different mechanisms used by PEDV to evade host innate immune responses.

## 2. Genomic Structure of PEDV

**Figure 1:**
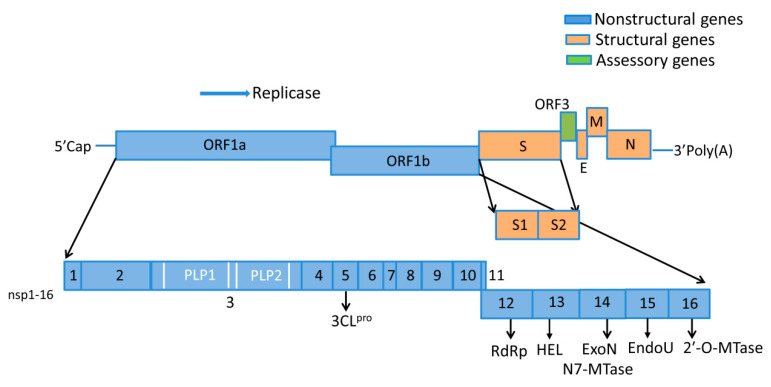
Porcine epidemic diarrhea virus (PEDV) genome constitution. The viral genome is composed of seven open reading frames (ORFs) and the 5′ and 3′ untranslated regions (UTRs). The ORFs encode four structure proteins (S, E, M and N), a hypothetical accessory protein (ORF3), as well as 16 nsps (nsp1–16). Nsp3 contains two papain-like proteases domains (PLP1 and PLP2). Definitions: 3CLpro, 3C-like protease (nsp5); RdRp, RNA-dependent RNA polymerase (nsp12); HEL, 5′-to-3′ Helicase (nsp13); ExoN, 3′-to-5′ exoribonuclease (nsp14); N7-MTase, N7-methyl transferase (nsp14); EndoU, endoribonuclease (nsp15); 2′-O-MTase, 2′-O-methyl transferase (nsp16).

PEDV is an enveloped, positive-sense, single-stranded RNA virus. The PEDV genome constitution represents a standard CoV arrangement. The viral genome is approximately 28 kb in length, containing a 5′ terminal cap, a 3′ poly (A) tail, as well as seven open reading frames (ORFs), including the ORF1a, ORF1b, S, ORF3, E, M, and N genes (Figure 1) [39,40,41]. The N terminal ORF1a and ORF1b encode two large replicase polyprotein precursors (pp1a and pp1ab), which are subsequently processed into 16 nonstructural proteins (nsp1 to 16). ORF1a encodes pp1a which is cleaved by viral proteases into 11 nonstructural proteins (nsp1-nsp11). The ORF1b generates five additional nonstructural proteins (nsp12–16) that are proteolytically cleaved by the viral proteases from pp1ab [42]. Nsp3 contains two papain-like protease domains (PLP1 and PLP2 or PL^pro^) that cleave the nsp1–4 region of the replicase polyprotein at three sites. Nsp5, a chymotrypsin-like enzyme also known as 3C-like protease, cleaves the polyprotein at remaining cleavage sites [43]. The C terminus of viral genome contains five ORFs, encoding four structure proteins (spike protein (S), small envelope glycoprotein (E), membrane glycoprotein (M), and nucleocapsid protein (N)), as well as a hypothetical accessory protein ORF3 [44,45,46]. The 16 nsps, together with the N protein, and several host proteins, form a large replication and transcription complex (RTC) that engages in the minus-strand RNA synthesis, using viral genomic RNA. These nsps play important roles in virion structure modification and the replication and transcription of PEDV [47]. 

## 3. Biological Functions of PEDV Proteins

The viral proteins of PEDV perform different biological functions during viral entry, replication cycle and propagation (Table 1). PEDV S protein, a type I membrane glycoprotein protein located on the envelope of the virus, consists of an N-terminal signal peptide, a large extracellular region, a single transmembrane domain, as well as a short cytoplasmic tail [48,49]. The ectodomain of S protein comprises S1 and S2 subunits. The N-terminal S1 region, containing N- and C-terminal domains (S1-NTD and S1-CTD), is mainly responsible for receptor binding [50]. The C-terminal membrane-anchored S2 region is mainly involved in triggering the fusion of the viral envelope with host cell membranes [48,49]. The interaction of the CoV S protein with its host cell surface receptor is a key determinant for host tropism. The S1-CTD of most known members of α-CoV genus, including PEDV, interacts with aminopeptidase N (APN) to entry into the target cell [32,36,51,52,53,54]. In addition, S protein contains the epitopes that are the major targets of the neutralizing antibody. N protein is the most abundant viral protein during the early phase of infection in CoV-infected cells [55]. Similar to the N proteins of other CoVs, the PEDV N protein has multiple functions, such as acting as a structural protein that forms nucleocapsid with viral genomic RNA, playing important roles in viral replication, transcription, and assembly [56,57]. The expression of the N protein in intestinal epithelial cells extends the S-phase of cell cycle, causes endoplasmic reticulum (ER) stress, and upregulates interleukin-8 expression [44]. Moreover, the N proteins of several *α*-CoVs and *β*-CoVs, including PEDV, PDCoV, SARS-CoV, and mouse hepatitis virus (MHV), have been identified as innate immunity antagonists [58,59,60,61]. However, the involved antagonistic mechanisms are particularly different. PEDV M protein participates in virion assembly and virus budding through collaboration with other viral proteins, and engages in the induction of neutralizing antibodies against PEDV [62,63]. PEDV M protein is distributed throughout the cytoplasm. It induces the cell growth retardation in intestinal epithelial cells (IEC) and arrests the cells in S-phase [64]. In addition, PEDV M protein is identified as an interferon (IFN) antagonist with an unrecognized mechanism [65]. PEDV E protein is important for the virus packaging and budding [66]. It is predominantly localized in the ER, having no effect on cell growth, cell cycle and cyclin A expression in IEC. However, it causes ER stress and activates the nuclear factor-κB (NF-κB) pathway which is responsible for the up-regulation of IL-8 and the anti-apoptotic protein Bcl-2 expression [67]. ORF3 has been predicted to possess multiple transmembrane domains [68], while it is predominantly distributed in the cytoplasm [69]. ORF3 also detains cells at S-phase, facilitating vesicle formation, and thus promoting PEDV multiplication [69]. A recent study suggests that ORF3 interacts with S protein during PEDV assembly and consequently benefits viral replication [70].

CoV nsps play multiple roles in the synthesis or processing of viral RNA, or in virus-host interactions aiming to create an optimal environment for virus replication, such as facilitating viral entry, viral gene expression, RNA synthesis, and virion release. nsp1 is a N-terminal cleavage product of ORF1a polyprotein [71], a 9-kDa protein, that exists only in α-CoVs and β-CoVs [72]. The nsp1 of *α*-CoVs is not very similar to *β*-CoVs nsp1 with regard to sequence homology and size [73,74]. Based on the sequence alignment analysis of the genomes of different CoVs, the viral nsp1 can be regarded as a genus-specific marker [75]. Moreover, *β*-CoVs nsp1 has been widely reported to inhibit host protein expression. However, the biological functions of *α*-CoVs nsp1 remain largely unknown. Despite the lack of overall sequence similarity, the nsp1 of different CoVs shares a similar function to interfere with host protein expression [76]. These studies suggest the importance of nsp1 in the life cycle of different lineages of CoVs. It is shown that TGEV nsp1 inhibits host gene expression and is critical for viral virulence [77]. PEDV nsp1 induces the degradation of CBP and NF-κB to abate IFN response [78], but the detailed mechanisms remain unclear. The sizes and amino acid sequence identity of nsp 2 are variable among different CoVs [76]. Nsp2 of MHV and SARS-CoV are involved in viral RNA synthesis [79]. PEDV nsp2 has unknown functions in replication, and may implicate the virus-host interactions and virulence. Nsp3 is the largest nsp protein, containing two papain-like protease (PLP1 and PLP2) domains, of which PEDV PLP2 acts as a viral deubiquitinase (DUB), to negatively regulate type I IFN signaling [80]. CoVs PLPs domains exhibit multiple functions, serving as a viral protease, DUB, as well as an IFN antagonist [81]. 

CoVs, like other positive-stranded RNA viruses, induce membranous rearrangements of varying morphologies that are essential for RTCs anchoring [82,83]. The CoV-induced replicative structures consist of double-membrane vesicles (DMVs) and convoluted membranes (CMs), which form a large reticulovesicular network that are critical for viral replication and transcription [84,85,86,87,88,89,90,91,92,93]. Among the CoV nsps, nsp3, nsp4, and nsp6 include the hydrophobic transmembrane domains engaging in anchoring the viral RNA synthesis components to the membranes [94]. For MERS-CoV and SARS-CoV, co-expression of nsp3 and nsp4 is required to induce DMVs [95]. SARS-CoV nsp6 has membrane proliferation ability as well, which also contributes to DMVs formation [96]. The structure and functions of α-CoV nsp3 are largely unknown [97]. Nsp4 is also a marker for CoV-induced membrane structures; some results indicate that the nsp4–10 of pp1a act as a large complex through multidomain structure or scaffold during viral RNA replication progress, before its cleavage into individual products [98]. CoVs nsp5 encodes a 3C-like proteinase (3CL^pro^). The polyproteins pp1a and pp1ab are processed into individual elements of replicase by 3C-like protease and PLPs [99]. Moreover, PEDV nsp5 plays a crucial role in virus replication and also blocks host innate immune responses [100]. Crystallographic or nuclear magnetic resonance structures have shown that nsp3, nsp5, nsp7, nsp8, nsp9, and nsp10 have the PLprob and the ADP-ribose 1′′-phosphatase (ADRP) activity [101,102,103,104,105,106,107]. The crystal structure of SARS-CoV nsp9 suggests that nsp9 is dimeric and it is able to bind to single-stranded RNA [108]. The crystal structure of SARS CoV nsp10 protein suggests that nsp10 is a zinc-finger protein, which is existent exclusively in CoVs so far [107]. Moreover, nsp7–10 have RNA binding activity and nsp12 encodes a single RNA-dependent RNA polymerase (RdRp). The biochemical characterization and crystallization of SARS CoV nsp7 and nsp8 manifests that eight copies of nsp8 and eight copies of nsp7 form a supercomplex [106]. The complex is supportive for nucleic acid binding and may be associated with the processivity of viral RdRp [102,106]. Recently, structural studies have described that the SARS-CoV nsp12 polymerase binds to the nsp7 and nsp8 complex [109] that may increase the polymerase activity of nsp12 RdRp [110]. CoV nsp13, a NTPase/helicase, is also determined to play essential roles in viral replication [111]. CoV nsp14 is a multifunctional protein with 3′-5′ exoribonuclease activity and N-7-methyltransferase [MTase] activity [112,113]. Nsp14 catalyzes the N7-methylation of Gppp-RNA to form a cap-0 structure. CoV nsp15 encodes an endoribonuclease (EndoU), performing functions through a hexamer in many CoVs [114,115,116]. The endoribonuclease activity of nsp15 is not essential for CoV replication [117,118]. For CoVs, the 5′ end of the viral genomic RNA and subgenomic mRNA (sgmRNA) is supposed to have cap structures: an N-7 methylated guanosine nucleoside (m7GpppN) (cap 0) and a methyl group at the 2′-*O*-ribose position (cap 1) of the first nucleotide [119]. These cap structures enhance the initiation of translation of viral proteins, protect viral mRNAs against cellular 5′-3′-exoribonuclease and limit the recognition of viral RNA by host innate system [120,121]. Nsp13 is proposed to catalyze the first step of the 5′-capping reaction of viral RNAs [122]. The methylation of the two sites in the 5′ cap are catalyzed by three nsps; nsp14 (the N-7-MTase), nsp16 (the 2′-O-methyltransferase), and nsp10 [112,123,124,125,126]. In addition, the 3′-5′ exoribonuclease activity of nsp14 is involved in a replicative mismatch repair system during RNA synthesis, which improves the replication fidelity of CoV [42]. Although these nsps have been demonstrated to play essential roles in viral replication, transcription and/or post-translational polyprotein processing [127], the nsp12–16 of PEDV and other CoVs are poorly characterized to date, except for SARS-CoV.

## 4. Innate Immunity during PEDV Infection

Host cells generally defend against virus infection by mounting an innate antiviral immune response to prevent the spread of the infection and aid in initiating an adaptive immune response which eventually removes the viruses from host. Therefore, the first barrier to restrain viral infections is the host innate immune system, which is related to multiple proteins and mechanisms, including IFNs, inflammatory cytokine, apoptosis, autophagy, and so on.

### 4.1. Overview of IFN Responses

The activation of type I IFN responses is composed of three stages: (1) recognition of pathogen-associated molecular pattern (PAMP) by PRRs; (2) secretion of type I IFNs through paracrine or autocrine pathways; and (3) expression of numerous antiviral IFN-stimulated genes (ISGs) which bring the host into the antiviral state [136]. At least three important PRRs have been identified in recognition of viral nucleic acids, including retinoic acid-inducible gene I (RIG-I)-like receptors (RLRs) (detection of viral RNA in the cytoplasm) [137], the membrane-bound Toll-like receptors (TLRs) (recognition of viral RNA or DNA in the endosome) [138], as well as a structurally unrelated group of viral DNA sensors (e.g., cGAS (cyclic GMP-AMP synthase) and IFI16) localized in the host cytoplasm and/or nucleus [139]. In the cytosol, the formation of specific secondary structure of viral RNA is closely related to viral RNA delivery and replication. These molecular signatures are detected by RLRs including RIG-I (also known as DDX58), melanoma differentiation-associated gene 5 (MDA5), and laboratory of genetics and physiology-2 (LGP2) [140,141]. PRRs recognize PAMPs, inducing an intracellular signaling cascade, thus leading to the activation of transcription factors such as IRFs and NF-κB, which in turn induce the production of IFNs [142]. Both RIG-I and MDA5 have two N-terminal CARD domains to interact with the CARD domains of the downstream adaptor proteins, and a DEAD/H-box RNA helicase domain for RNA binding. However, LGP2 does not have the N-terminalCARD domains, and the involved functions remain unclear [143,144,145]. dsRNA, as a specific secondary structure of viral RNA, can be sensed by RIG-I/MDA5 to induce IFN-α/β production through the cascade activation of the RLR pathway [146,147,148]. Activated RIG-I/MDA5 forms homo-oligomers and recruits the adaptor mitochondrial antiviral signaling (MAVS) to induce the formation of signaling complex of MAVS with other proteins on mitochondria. TNF receptor-associated factor 6 (TRAF6) associates with TNFR1-associated death domain protein (TRADD), tripartite motif 14 (TRIM14), and pyruvate carboxylase (PC), resulting in the activation of IκB kinases (IKKs). IKKs (IKKα, IKKβ and IKKγ) phosphorylate NF-κB inhibitor (IκB), leading to the ubiquitination of IκB and its subsequent degradation. NF-κB is then activated and translocated into the nucleus to turn on the expression of proinflammatory and type I IFN. MAVS also recruits and activates TBK1/IKKε by TRAFs that are pre-associated with TBK1/IKKε, via direct interaction between the SDD domain of TBK1/IKKε and the coiled-coil domain of TRAFs [149]. Activated TBK1/IKKε phosphorylates IFN regulatory factors (IRF3/IRF7), that further dimerize and import into the nucleus to promote type I IFN production. On the other hand, MAVS interacts with MITA (also known as STING), that is located in mitochondria and the endoplasmic reticulum (ER) membrane. MITA interacts with the TRAP complex, which may be involved in recruiting TBK1 and IKKε to phosphorylate IRF3.

Ubiquitination and deubiquitination are decisive in the regulation of RLR pathways activation [150]. Upon binding to viral dsRNA, RIG-I and MDA5 undergo conformational changes and release the N-terminal tandem CARD domains [151,152,153]. The CARD domains of RIG-I are modified by lysine 63 (K63) polyubiquitin chains through the ubiquitin ligases TRIM25, RNF135, and RIPLET. This modification is crucial for RIG-I to recruit MAVS [154,155]. In addition to the ubiquitination of RLRs, the polyubiquitylation of TRAF3 and TRAF6 also play an important role in the regulation of innate immune signaling by activation of TBK1 and IKKs, respectively. The K63 polyubiquitin chains can be removed by DUBs such as the tumor suppressor protein CYLD, DUBA and A20, providing a mechanism to downregulate immune responses [156]. Ubiquitination and deubiquitination are in a dynamic equilibrium to maintain immune homeostasis.

Type I IFNs are secreted by secretory cells and peripheral cells through self-secretion or paracrine secretion manners. Extracellular type I IFNs bind to a heterodimeric complex composed of subunits of IFN-α receptors 1 (IFNAR1) and 2 (IFNAR2) located on the cell surface, which activates the tyrosine kinase 2 (TYK2)/Janus kinase 1 (JAK1) signal transducer. TYK2/JAK1 subsequently induces the phosphorylation of transcription factors STAT1 and STAT2, which dimerize and in turn recruit IFN-regulatory factor 9 (IRF9), to form STAT1-STAT2-IRF9 trimerized complex (ISGF3). This complex then translocates to the nucleus, where it binds to the IFN-stimulated response elements (ISRE motif; conserved sequence is TTTCNNTTTC) [157]. The binding of ISGF3 to ISRE finally triggers expression of IFN-stimulated genes (ISGs) that directly or indirectly exert antiviral effects in host cells [157].

Three types of IFNs (types I, II and III) have been identified. Type I and type II IFNs have been widely reported. Type II IFNs only contain IFN-γ. IFN-γ is produced by natural killer (NK) cells and activated CD4^+^ and CD8^+^ T cells in response to the cytokines such as interleukin-12 (IL-12) and IL-18 [158]. IFN-γ binds to the type II IFN receptor composed of two subunits, IFNGR1 and IFNGR2. IFNGR1 and IFNGR2 induce the formation of STAT1-STAT1 homodimers. STAT1-STAT1 homodimers translocate to the nucleus and bind to the promoter of the IFN-γ-activation site (GAS) elements, to initiate the transcription of IFN-γ-regulated genes [159]. 

Type III IFNs have been explored in recent years, to unravel the underlying mechanisms that manipulate host innate immune responses. Type III IFNs in humans contains IFN-λ1 (interleukin 29 [IL-29]), IFN-λ2 (IL-28A), IFN-λ3 (IL-28B), and IFN-λ4 [160,161,162]. Their expression profiles, signaling pathways, and gene expression programs resemble those of type I IFNs. The production of type I and III IFNs are both initiated through the recognition of PAMPs or damage associated molecular patterns (DAMPs) by PRRs [163]. Despite the fact that the same transcriptional factors are required for the activation of promoters of type I and III IFNs, the NF-κB pathway is a pivotal regulator in IFN-λ production, whereas the IRFs pathway dominates type I IFNs expression. The promoter of IFN-λ1 includes more NF-κB binding sites compared with in the IFN-β promoter [164]. 

The signal transduction of type III IFNs depends on the IFN-λ-specific receptor, IL-28Ra chain and IL-10R2 chain [161,165]. Synthetic IFN-λ binds to IL-28Rα and induces a conformational change within the receptor subunits, that triggers the activation of the receptor-associated tyrosine kinases (TYK2 and JAK1), which then phosphorylate STAT1 and STAT2. STAT1 and STAT2 are heterodimerized and interact with IRF9 to form the ISGF3 transcription complex that binds to ISRE in the promoters of ISGs, to induce the expression of hundreds of proteins with antiviral functions [166].

### 4.2. Immune Evasion Mechanisms of PEDV 

Induction of IFN-α/β is the most rapid and effective mechanism for hosts to initiate antiviral innate immune responses. SARS-CoV, MHV and many other CoVs are sensitive to IFNs. A great number of viral dsRNAs intermediates are generated during CoVs infection that contribute to IFN production, but these CoVs remain highly pathogenic. As a matter of fact, CoVs have developed a set of elaborate mechanisms to evade or inhibit the host antiviral innate immune response during virus evolution [134,167]. The evasive strategies utilized by PEDV are classified into four major types: (1) inhibition of RLRs-mediated IFN production pathways, (2) inhibition of the activation of transcription factors responsible for IFN induction, (3) disruption of the signal cascades induced by IFN, and (4) hiding its viral RNA to avoid the exposure of viral RNA to immune sensors. In the past decade, accumulating evidence demonstrates that PEDV N protein, nsp1, PLP2, nsp5, nsp15, and nsp16 antagonize type I IFN or type III IFNs production [58,65,78,80,100,132,135,168]. This explains why only weak IFNs’ and cytokines’ expression is detected in PEDV-infected cells [169,170]. 

#### 4.2.1. PEDV N Protein

N protein, as an abundantly produced structural protein within CoV-infected cells, has multiple functions, including virus replication, transcription, and assembly [56,134]. PEDV N protein has been identified as an IFN antagonist that blocks the expression of IFN-β and ISGs by suppression of the IRF3 and NF-κB activities [58]. PEDV N protein inhibits the activation of the IFN-β promoter induced by TBK1 and its upstream RIG-I, MDA-5, VISA, and TRAF3, while not affecting the activation of the IFN-β promoter driven by IRF3. Further experiments confirm that N directly interacts with TBK1 to obstruct the association between TBK1 and IRF3, which inhibits TBK1-induced IRF3 phosphorylation and IFN-β production [58]. Moreover, the effect of PEDV N protein on type III IFN production has also been evaluated [168]. N protein inhibits polyinosinic-polycytidylic acid (poly(I:C))-induced IFN-λ3 production by blocking the nuclear translocation of NF-κB, but does not antagonize the type I or type II IFN expression induced by poly(I:C) in IPEC-J2 cells [168].

Recent studies show that SARS-CoV N protein inhibits type I IFN production through suppressing TRIM25-mediated RIG-I ubiquitination [171]. The MERS-CoV N protein also blocks IFN production by interacting with TRIM25 [171]. In addition, both MHV and SARS-CoV N proteins perturb the function of cellular protein activator of protein kinase R (PACT), which can bind to RIG-I and MDA5 to activate IFN production, and thus antagonize type-I IFN signaling [61]. These results indicate the important function of the CoVs N protein in modulating host innate immune response. Whether PEDV N protein targets TRIM25 or PACT should be investigated.

Although several studies have been performed to understand the pathogenicity of PEDV, there remains limited information about the interaction between viral proteins and host cell factors during viral infection. CoV N protein is a vital viral protein involved in virus replication. Current researches have indicated that N protein interacts with many host proteins, such as hCypA [172], proteasome subunit p42 [173], Smad3 [174], hnRNP-A1 [175], and the chemokine CXCL16 [176]. In the host cells, a large number of host proteins reveal various functions. However, for the virus, the genome only encodes several limited viral proteins. Therefore, these viral proteins have to be multifunctional, which is pivotal to virus replication and existence. 

#### 4.2.2. PEDV nsp1 

PEDV nsp1 is the N-terminal cleavage product from polyproteins pp1a and pp1a/b processed by nsp3 and nsp5 [177] and is about 110 amino acids in length [74,178]. Nsp1 of many α-CoV and β-CoV exhibits both functional conservation and mechanistic diversity in suppressing host gene expression and IFN signaling. For SARS-CoV, nsp1 triggers the decay and cleavage of host mRNA and inhibits host protein translation, subsequently inhibiting type I IFN production [179,180]. SARS-CoV nsp1 also blocks the expression of IFN-inducible genes, by restraining the signal transduction during virus infection [181,182]. The TGEV nsp1 considerably suppresses host protein expression during viral infection [77]. Structural studies show that the core structure of PEDV nsp1 is highly similar to those of SARS-CoV nsp1 and TGEV nsp1 [183]. PEDV nsp1 inhibits host gene expression and three motifs (amino acids 67 to 71, 78 to 85, and 103 to 110) form a stable functional region for inhibition of host protein synthesis, differing considerably from SARS-CoV nsp1 [183]. 

PEDV nsp1 has been identified as an IFN antagonist, which constrains poly (I:C)-induced IFN-β promoter activity [65]. Nsp1 significantly inhibits the activation of IFN-β promoter triggered by IRF3, whereas it does not inhibit IRF3 phosphorylation and its nuclear translocation. Nsp1 interrupts the association of IRF3 with CREB-binding protein (CBP), by promoting CBP degradation in the nucleus via the proteasome-dependent pathway. CBP/p300, the transcription co-activator cAMP responsive element binding protein (CREB), forms a complex with the activated IRF3 in nucleus. The IRF3-CBP/p300 complex binds to the positive regulatory domain (PRD) regions of the IFN-β promoter, to assemble the enhanceosome with NF-κB and other factors, which ultimately turn on the transcription of type I IFN genes [184,185,186]. Therefore, PEDV nsp1 blocks type I IFN production in the nucleus. Activated NF-κB induces the production of type I IFNs and proinflammatory cytokines and is important for inhibiting viral infection. PEDV nsp1 has been shown to interfere with the NF-κB activity [78] and is the most potent suppressor of proinflammatory cytokines at early infection. It inhibits the phosphorylation and degradation of IκBα, and blocks p65 nuclear translocation, leading to the suppression of both IFN and the early production of pro-inflammatory cytokines [78]. Moreover, PEDV inhibits type III IFN production and nsp1, nsp3, nsp5, nsp8, nsp14, nsp15, nsp16, ORF3, E, M, and N are identified as type III IFN antagonists. Among these antagonists, nsp1 is the most potent suppressor [130]. PEDV nsp1 blocks the nuclear translocation of IRF1 and decreases the amounts of peroxisomes and then suppresses IRF1-mediated type III IFNs. The conserved residues of PEDV nsp1 protein are crucial for IFN suppression [130]. Multiple effects of nsp1 on modulating innate immune response during PEDV infection suggest the vital role of nsp1 in the PEDV replication cycle. 

#### 4.2.3. PLP2

The antiviral innate immune signaling pathways are regulated by several posttranslational modifications (PTMs), such as phosphorylation, ubiquitination, glycosylation, NEDDylation and SUMOylation [187], of which ubiquitination is a critical modification to modulate the stability and activity of PRRs and other components of innate immune signaling pathways. During viral infection, a reciprocatory action (occurrence of ubiquitination and deubiquitination) helps maintain the homeostasis of host immune responses. Hence, deubiquitinases (DUBs) are indispensable in the regulation of virus-induced type I IFN signaling [188]. Many host DUBs have been reported engaging in the regulation of innate immune signaling pathways [189,190,191]. In recent years, a variety of viral DUBs have been discovered to target key components of type I IFN pathway during various RNA virus infections. For example, foot-and-mouth disease virus leader proteinase (FMDV Lb^pro^) [192], and porcine reproductive and respiratory syndrome virus nsp2 (PRRSV nsp2) possess ubiquitin-deconjugating activity to deubiquitinate key host components [193,194]. To counteract host antiviral response, CoVs likely take advantage of DUB activity to break host innate immunity. Indeed, the PLPs of mouse hepatitis virus A59 (MHV-A59) [195], SARS [196], and human CoV NL63 have DUB activity and antagonize IFN induction [197]. PEDV PLP2 has been reported as having a deubiquitinase activity as well, and it can be co-immunoprecipitated by RIG-I and STING. As mentioned above, FMDV Lb^pro^, MHV PLP2 and SARS PLPs all counteract host innate immune response through blocking the ubiquitination of the components of RLRs pathways. Similarly, PEDV PLP2 removes the ubiquitinated conjugates from RIG-I and STING by its DUB activity, to negatively regulate type I IFN production. PEDV PLP2 probably interacts with RIG-I and STING, which prevents the activation of RIG-I and STING by hindering the recruitment of downstream signaling molecules. As expected, the interference with the ubiquitination of RIG-I and STING by PLP2 clearly benefits PEDV replication [80]. PEDV nsp3 contains two core domains of PLPs (PLPl and PLP2). It is determined that PEDV PLP2, but not PLP1, inhibits the IFN-β promoter activation in HEK293T cells. The DUB activity of PLP2 is highly dependent on its catalytic activity. Three catalytically inactive mutants of PEDV PLP2 (C1729A, H1888A and D1901A) are defective in the deubiquitination of its targets and fail to impair virus-induced IFN-β production.

SARS-CoV PLP2 interacts with MDM2 (mouse double minute 2 homolog) to deubiquitinate and stabilize MDM2, approving the degradation of p53 and the suppression of IFN signaling [198]. PEDV infection degrades p53 by upregulation of MDM2 expression [198]. PEDV PLP2 may be responsible for targeting the p53 pathway and inhibiting p53-dependent apoptosis, leading to immune evasion. A recent study determined that TGEV PL1 inhibits the IFN-β expression and interferes with the RIG-1- and STING-mediated signaling pathway through a viral DUB activity [195]. It suggests that different viral proteins are involved in the deubiquitination of host proteins for different CoVs. However, these studies offer a probability to design a common therapeutic against different viral DUBs to reduce the replication and pathogenesis of CoVs. Therefore, further studies are required to understand more about the substrate specificity of these viral DUBs and clarify the precise functions of CoV protease/DUB activity.

#### 4.2.4. PEDV nsp5

Notably, 3C^pro^ is a critical IFN antagonistic protein identified in multiple different families of viruses. The 3C^pro^ of picornaviruses, including FMDV [199], hepatitis A virus (HAV) [200], enteroviruses (EV71, EV-D68) and coxsackieviruses (CVB3, CV-A16, CV-A6) [201,202,203,204], antagonize innate immune signaling by targeting the critical components of the IFN pathways for proteolysis. A newly emerged picornavirus, Seneca Valley virus (SVV), has also evolved an effective mechanism to escape host antiviral innate immune using its 3C^pro^. Moreover, 3C^pro^ cleaves the signaling components (MAVS, TRIF, and TANK) of type I IFN pathway and induces the degradation of the transcription factors IRF3 and IRF7 to constrain host antiviral response [205,206]. 

CoV nsp5 is called 3C-like protease (3CL^pro^), that resembles the 3C^pro^ of other RNA viruses. For CoV, the polyprotein precursors (pp1a and pp1b) are mainly processed to generate mature nonstructural proteins by 3CL^pro^. To date, the 3CL^pro^ of CoVs, including PEDV and PDCoV, have been confirmed to antagonize type I IFN production by the cleavage of NF-κB essential modulator (NEMO) and STAT2 [100,207,208]. NEMO is essential for RNA virus-induced activation of NF-κB, IRF3, and IRF7 [209]. NEMO is required for MAVS-induced IKKα/β activation and is also crucial for the activation of TBK1/IKKε [149]. To establish successful infections, PEDV targets NEMO to subvert host innate immune responses. PEDV nsp5 significantly inhibits Sendai virus (SeV)-induced IFN-β synthesis and the process depends on its protease activity [100]. Further experiments show that PEDV nsp5 inhibits RIG-I/MDA5 signaling and targets the upstream of TBK1. The cleavage of NEMO by nsp5 is identified as responsible for this inhibitive effect. The PEDV nsp5-mediated cleavage of NEMO efficiently blocks NEMO-mediated downstream signaling. The cleavage site within NEMO that is grasped by nsp5 has been determined. Of these reported immune evasion strategies employed by CoVs, the cleavage of innate immune adaptors is a particularly effective manner to disrupt antiviral responses. Nsp5 is essential for the life cycle of PEDV and other CoVs [210,211]. It is a potential target for the development of anti-coronaviral therapeutics. Although PEDV nsp5 does not target STAT2 mediated type Ⅰ IFN signaling pathway, PEDV nsp7 has been reported to inhibit the STAT1 and STAT2 induced activation of ISRE [212]. Nsp7 competes with Karyopherin α (KPNA1), which is an adaptor mediating nuclear translocation of ISGF3, in combination with STAT1, to block ISGF3 nuclear transport. However, the expression and phosphorylation of STAT1 and STAT2 are not affected by PEDV nsp7. In fact, PEDV infection degrades STAT1, leading to the inhibition of IFN signaling [170]. Therefore, other PEDV encoded proteins likely target IFNs mediated signaling.

#### 4.2.5. Evasion of Viral RNA Recognition

**Figure 2:**
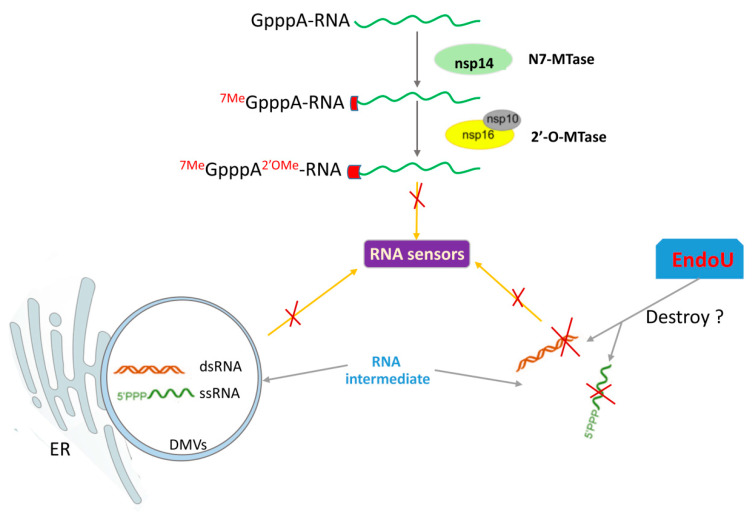
The model of PEDV to avoid viral RNA recognition by RNA sensors. The immunosuppressive effect of PEDV nsp16 and nsp15 suggests that PEDV 5’ mRNA capping and viral EndoU activity are important for innate immune evasion. The formation of double-membrane vesicles (DMVs) induced by viral infection is also an important strategy for hiding of RNA intermediates.

CoVs belong to RNA viruses, which produce several RNA species, such as dsRNA intermediates and RNA with a 5′-triphosphate during replication. These RNA intermediates are potent stimulators of PRRs and are associated with the organelles of viral RNA replication, DMVs [213,214]. DMVs formed from membranous rearrangements seem to sequester the replication intermediates using membrane-bound vesicles or invaginations to keep away from PRRs. Therefore, the form of DMVs may be a strategy for PEDV to escape innate immune recognition in the cytosol (Figure 2). However, whether DMVs alone are sufficient to shield RNA from PRRs remains unknown. Besides, the replication organelles, the endoribonuclease activity and viral 5′ end RNA capping/protection mechanisms are also the critical ways of avoiding RNA recognition or protecting it from degradation [132,135]. 

1) PEDV nsp15

CoV nsp15 has EndoU catalytic activity that was initially thought to play a vital role in virus replication. However, the catalytic-defective EndoU of MHV shows only a subtle defect in viral replication compared to WT virus in fibroblasts [215]. Similar results are found for the nsp15 mutants of SARS-CoV and HCoV-229E [216]. These findings suggest that the EndoU activity of nsp15 is not required for RNA synthesis. Recently, nsp15 has been demonstrated to act as a new IFN antagonist of CoVs [117,118,217]. Recent reports indicate that CoVs’ EndoU activity is essential for prevention of RNA recognition by MDA5, protein kinase R (PKR), and OAS/RNAse L system [118]. PKR and OAS/RNAse L recognize and destroy foreign RNA in the cytosol to defend viral infections. To counteract the function of PKR and OAS/RNAse L, the virus hides or modifies its viral RNA, to avoid the exposure of viral RNA to these molecules. In all CoVs, the EndoU catalytic domain in nsp15 is highly conserved. PEDV EndoU activity has been indicated as having an antagonistic effect on IFN signaling [135]. The EndoU activity of PEDV nsp15 not only inhibits the type I IFN response in porcine macrophages, but also antagonizes the type III IFN response in porcine epithelial cells. The replication of EndoU-mutant PEDV (icPEDV-EnUmt) is considerably impaired in porcine epithelial cells compared to the wild type PEDV (icPEDV-wt). The icPEDV-EnUmt clearly induces early and robust type I and type III IFNs production, as well as ISGs’ expression compared with that induced by icPEDV-wt. The EndoU-deficient PEDV infected animals also show reduced viral shedding and mortality. These results indicate that the EndoU activity of PEDV nsp15 plays a vital role in evading host antiviral innate immunity (Figure 2) [135].

2) PEDV nsp16

CoV nsp16 is a 2′-*O*-methyltransferase (2′-*O*-MTase). To evade recognition by the host immune sensors, many CoVs encode methyltransferases involved in the capping of viral RNA. This modification makes the viral RNA indistinguishable with host cell mRNA, which is important to avoid the recognition of viral RNA by MDA5 (Figure 2) [121,218]. Methylation of the two sites in the 5′ cap is catalyzed by three nsps, including nsp14 (the N-7-MTase), nsp16 (the 2′-*O*-MTase), and nsp10 [112,123,124,125,126]. For example, SARS-CoV nsp16 acts as a 2′-*O*-MTase to prevent innate immune recognition and promote viral proliferation [113,121,123]. PEDV nsp14 and nsp16 have been identified as the viral IFN antagonists [65,132]. The overexpression of nsp14 or nsp16 remarkably inhibits IFN-β production, but nsp16 appears to play a more important role in innate immunity regulation than nsp14 [132]. Nsp16 is a highly conserved methyltransferase which contains an invariant KDKE motif within the methyltransferase core [219]. This KDKE motif is required to mediate its activity. Notably, the mutation of any of the KDKE active sit has been shown to abolish the 2′-*O*-MTase activity [123]. PEDV nsp16 KDKE motif plays a critical role in the inhibition of type I IFN production, suggesting the important role of the 2′-*O*-MTase in PEDV-mediated immune evasion. PEDV nsp16 negatively regulates RLR-mediated signal pathway activation, and inhibits the expression of the IFN-stimulated IFIT family members (IFIT1, IFIT2, IFIT3), which in turn promotes PEDV replication. Taken together, these results demonstrate that PEDV nsp16 negatively regulates cellular antiviral response to promote viral replication [132]. Screening inhibitors targeting the 2′-*O*-MTase of nsp16 might be a prominent strategy to inhibit CoV infections and develop antivirals for treatment of the diseases caused by CoVs. Additionally, CoVs nsp14 also includes the 3′-to-5′ exoribonuclease (ExoN) activity [113]. A mutation of TGEV nsp14 ExoN generates lower levels of dsRNA than wildtype TGEV and thus triggers a reduced antiviral response [220]. Nsp14 ExoN activity is also critical for the resistance of host innate immune response in MHV-infected cells [221]. The role of nsp ExoN activity of PEDV in counteracting host antiviral response should be investigated to uncover more functions of PEDV nsps. These data suggest that PEDV has evolved multiple evasive mechanisms to circumvent viral RNA recognition or prevent RNA degradation to establish a successful infection in the host. 

#### 4.2.6. PEDV Inhibits Heat Shock Protein 27 Expression to Impair Innate Immune Signaling

Heat shock protein 27 (HSP27) belongs to the small heat shock proteins family, which has been identified as a multifunctional protein involved in cytoskeletal stability, proinflammatory processes, and the inhibition of apoptosis [222,223]. Several HSPs have been reported to be implicated in PEDV infection in vitro and in vivo [224,225]. Indeed, the infection of many viruses up-regulates HSP27 expression by different mechanisms to delay cellular apoptosis, then supplies sufficient time for viral replication [226,227]. However, PEDV infection significantly induces the decreased expression of HSP27 in Vero cells [225] and MARC-145 cells [228]. HSP27 activates the phosphorylation of NF-κB, and thus promotes the mRNA expression of IFN-β in MARC-145 cells. As HSP27 is an upstream regulator of antiviral immune signaling, overexpression of HSP27 significantly inhibits the PEDV replication. PEDV has developed a strategy via mediating the suppression of HSP27 production to escape from host innate immune response [228]. HSP70, the most conserved HSP, is also important for the multiplication of several CoVs. The recruitment of HSP70 is thought to be a viral survival strategy for several viruses in their hosts [229]. The relationship between HSP70 and PEDV should be exploited further in future.

### 4.3. Modulation of Apoptosis

Viral infection triggers host immune response to induce IFNs’ and inflammatory cytokines’ production. Released IFNs elicit the expression of numerous ISGs which limit viral replication in the infected cells. However, the release of excessive amounts of IFNs and inflammatory cytokines will lead to autoimmune and auto-inflammatory diseases. The concomitant uncontrolled apoptosis is also one outcome that is harmful to the host. To maintain the reaction in a proper balance, hosts have evolved a series of effective mechanisms to control the antiviral innate immune response [230]. In contrast, viruses often break this balance, causing improper apoptosis reaction, which benefits viral replication.

PEDV infects various host cells including Vero, PK-15 and Marc-145 and cause obvious cytopathic effects. PEDV-induced apoptosis of the infected cell has been demonstrated both in vitro and in vivo [231]. Apoptosis is induced through the activation of apoptotic caspases, including caspase-2, -3, -6, -7, -8, -9, and -10 [232]. PEDV infection results in obvious caspase-3 and caspase-8 activation, as well as the cleavage of apoptosis-inducing factor mitochondria-associated 1 (AIFM1) and poly ADP-ribose polymerase (PARP), which leads to apoptotic nuclear fragmentation. PEDV spike protein S1 significantly elicits host cell apoptosis, while the nsp1–16 and other structural proteins (M, N, E, S2, and ORF3) have none or few effects on cell apoptosis. Therefore, S1 protein is probably the critical protein mediating the apoptosis induced by PEDV [128]. 

### 4.4. ER Stress

The multiple stages of CoV replication cycle are closely associated with cellular membrane compartments, especially the endoplasmic reticulum (ER). The shape and functions of ER can be influenced by different physiological states and environmental conditions. When protein synthesis amounts surpass the folding capacity, the accumulation of a large amount of unfolded proteins in the ER leads to ER stress. Consequently, cells manifest a corresponding biological reaction that is widely known as the unfolded protein response (UPR) [233]. Once the UPR is induced, it alleviates the problems by host protein translation inhibition (by the transducer PKR-like ER protein kinase (PERK)-induced phosphorylation of eIF2a), stimulating protein folding. If homeostasis cannot be re-established, apoptosis eventually is triggered. Indeed, the activation of UPR regulates a wide variety of signaling pathways, such as apoptosis, autophagy, mitogen-activated protein (MAP) kinase activation, and innate immune response [234].

Furthermore, *α*-CoV and *β*-CoV may induce ER stress in the infected cells [235]. PEDV ORF3, as the only accessory protein encoded by PEDV, is thought to be related to virus production and virulence of PEDV [68]. A series of studies suggest that ORF3 plays multiple roles, in addition to acting as an ion channel during PEDV replication. Recent studies show that PEDV ORF3 consists of four transmembrane domains (TMDs) and localizes in the cytoplasm in the aggregation manner [236]. ORF3 is a transmembrane protein, and the confocal microscopy analysis indicates that the aggregated ORF3 localizes in the ER to induce the ER stress associated with either apoptosis or autophagy. However, PEDV ORF3 induces the autophagy via driving conversion of LC3-I to LC3-II, but not influencing the apoptosis. ORF3-induced autophagy is dependent on ER stress response. PEDV ORF3 triggers ER stress response via the up-regulation of GRP78 protein expression and the activation of the PERK-eIF2α signaling pathway. Moreover, ORF3 protein is identified as an IFN antagonist to block IFN response by an unknown mechanism in PEDV-infected cells [65]. The functions of PEDV ORF3 should be further exploited.

## 5. Conclusions

**Figure 3:**
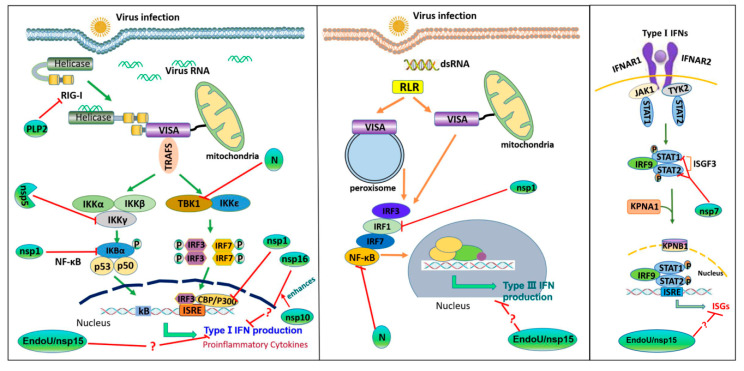
The action model of PEDV interferons (IFNs) antagonists. For type I IFN, PEDV PLP2 removes ubiquitinated conjugates from RIG-I; PEDV nsp5 induces cleavage of NEMO; PEDV N protein directly interacts with TBK1 to obstruct the association between TBK1 and IRF3; PEDV nsp1 causes degradation of CBP and IκBα, as well as inhibition of IκBα phosphorylation and p65 activation. PEDV nsp16 inhibits type I IFN production and nsp10 enhances the inhibitory effect of nsp16 on type I IFN production. For type III IFN, PEDV N protein blocks the nuclear translocation of NF-κB; PEDV nsp1 blocks the nuclear translocation of IRF1 and reduces the amounts of peroxisomes. PEDV nsp15 inhibits the type I IFN and type III IFN responses by unknown mechanisms. PEDV nsp7 interacts with STAT1 and STAT2 to block nuclear translocation of ISGF3.

PEDV has caused epidemic and endemic infections in pig populations in many countries and has become a major economic threat to the swine industry. Previous studies have identified viral factors that target key signaling molecules in the RLRs’ pathways, as well as viral factors that target the downstream signaling pathways responsible for ISGs induction. Of the 23 PEDV-encoded proteins, at least 10 viral proteins have been identified as type I IFN antagonists [65,78]. The mechanisms utilized by PEDV nsp1, PLP2, nsp5, and N protein to antagonize type I IFN production have been clarified (Figure 3, [58,65,78,80,129]). However, the specific mechanisms of other viral proteins to inhibit type I IFN production remain largely unknown. At present, 11 PEDV proteins have been identified as type III IFNs’ antagonists. The suppression of type III IFN signaling by N protein, nsp1, as well as nsp15, has been reported, while the mechanisms utilized by these viral proteins need to be further investigated. In addition, the CoVs replication cycle may induce the changes of ER stress, cell apoptosis, autophagy pathways, which contain intricate virus-host interactions and cross-talk relationships. Thus, more researches for PEDV are needed to truly reflect viral evasions from innate immune defenses. The findings of PEDV-host interactions will help prevent and control PEDV spreading.

## Figures and Tables

**Table 1:** The functions of PEDV viral proteins.

PEDV Proteins	The Biological Processes Involved	References
**S (S1 and S2)**	receptor binding and subsequent membrane fusion; replication cycle and propagation; cell apoptosis induction	[48,49,50,128]
**N**	viral replication, transcription, and assembly; extension of S-phase of cell cycle; endoplasmic reticulum (ER) stress formation; innate immunity antagonist; interaction with host protein NPM1	[44,56,57,129]
**M**	virion assembly and virus budding; induction of PEDV neutralizing antibodies; cell growth retardation in S-phase; IFN antagonist with unknown mechanism	[62,63,64,65]
**E**	virus packaging and budding; stimulation of ER stress and activation of NF-κB pathway	[66,67]
**ORF3**	detention of cells at S-phase; facilitating vesicle formation; promoting PEDV replication	[69,70]
**nsp1**	type I IFNs and type III IFN antagonists; inducing the degradation of CBP; suppression of phosphorylation and degradation of IκBα, as well as p65 nuclear translocation; blocking nuclear translocation of IRF1	[65,78,130]
**nsp2**	Unknown	
**nsp3,4,6**	nsp3, 4, and 6 are all involved in viral replication process; nsp3 also acts as a viral deubiquitinase (DUB) that deubiquitinates RIG-I and STING and negatively regulates type I IFN signaling	[80,94,99]
**nsp5**	3CL^pro^; processing replicase polyprotein during virus replication; blocking host innate immune responses by cleavage of NF-κB essential modulator (NEMO)	[99]
**nsp7–10**	Nsp7 is an IFN antagonist; nsp8 inhibits type III IFN response; nsp9 is involved in nucleic acid binding; nsp10 enhances the inhibitory effect of nsp16 on IFN-β production.	[65,131,132,133]
**nsp12**	encoding a RdRp; viral replication	[134]
**nsp13**	a NTPase/helicase that is essential for viral replication	[111]
**nsp14**	N-7-MTase; catalyzing N7-methylation of Gppp-RNA to form a cap-0 structure; 3′-5′ exoribonuclease activity involves in a replicative mismatch repair system during RNA synthesis; IFN antagonist	[42,65,112,113,119,133]
**nsp15**	EndoU; IFN antagonist; inhibition of the type I IFN and the type III IFN responses	[114,115,116,135]
**nsp16**	2′-*O*-MTase that catalyzes methylation of the 5’ cap; IFN antagonist	[112,123,124,125,126,132]

## References

[1] Sun R.Q., Cai R.J., Chen Y.Q., Liang P.S., Chen D.K., Song C.X. (2012). Outbreak of porcine epidemic diarrhea in suckling piglets, China. Emerg. Infect. Dis..

[2] Wang D., Fang L., Xiao S. (2016). Porcine epidemic diarrhea in China. Virus Res..

[3] Debouck P., Pensaert M. (1980). Experimental infection of pigs with a new porcine enteric coronavirus, CV 777. Am. J. Vet. Res..

[4] Wood E.N. (1977). An apparently new syndrome of porcine epidemic diarrhoea. Vet. Rec..

[5] Chasey D., Cartwright S.F. (1978). Virus-like particles associated with porcine epidemic diarrhoea. Res. Vet. Sci..

[6] Pensaert M.B., de Bouck P. (1978). A new coronavirus-like particle associated with diarrhea in swine. Arch. Virol..

[7] Li W., Li H., Liu Y., Pan Y., Deng F., Song Y., Tang X., He Q. (2012). New variants of porcine epidemic diarrhea virus, China, 2011. Emerg. Infect. Dis..

[8] Wang X.M., Niu B.-b., Yan H., Gao D.-s., Yang X. (2013). Genetic properties of endemic Chinese porcine epidemic diarrhea virus strains isolated since 2010. Arch. Virol..

[9] Sun M., Ma J., Wang Y., Wang M., Song W., Zhang W., Lu C., Yao H. (2015). Genomic and epidemiological characteristics provide new insights into the phylogeographical and spatiotemporal spread of porcine epidemic diarrhea virus in Asia. J. Clin. Microbiol..

[10] Stevenson G.W., Hoang H., Schwartz K.J., Burrough E.R., Sun D., Madson D., Cooper V.L., Pillatzki A., Gauger P., Schmitt B.J. (2013). Emergence of Porcine epidemic diarrhea virus in the United States: Clinical signs, lesions, and viral genomic sequences. J. Vet. Diagn. Invest..

[11] Alvarez J., Sarradell J., Morrison R., Perez A. (2015). Impact of porcine epidemic diarrhea on performance of growing pigs. PLoS ONE.

[12] Fan H., Zhang J., Ye Y., Tong T., Xie K., Liao M. (2012). Complete genome sequence of a novel porcine epidemic diarrhea virus in south China. J. Virol..

[13] Li R., Qiao S., Yang Y., Su Y., Zhao P., Zhou E., Zhang G. (2014). Phylogenetic analysis of porcine epidemic diarrhea virus (PEDV) field strains in central China based on the ORF3 gene and the main neutralization epitopes. Arch. Virol..

[14] Chen N., Li S., Zhou R., Zhu M., He S., Ye M., Huang Y., Zhu C., Xia P., Zhu J. (2017). Two novel porcine epidemic diarrhea virus (PEDV) recombinants from a natural recombinant and distinct subtypes of PEDV variants. Virus Res..

[15] Song D.S., Oh J.S., Kang B.K., Yang J.S., Moon H.J., Yoo H.S., Jang Y.S., Park B.K. (2007). Oral efficacy of Vero cell attenuated porcine epidemic diarrhea virus DR13 strain. Res. Vet. Sci..

[16] Song D., Moon H., Kang B. (2015). Porcine epidemic diarrhea: A review of current epidemiology and available vaccines. Clin. Exp. Vaccine Res..

[17] Wang X.N., Wang L., Zheng D.Z., Chen S., Shi W., Qiao X.Y., Jiang Y.P., Tang L.J., Xu Y.G., Li Y.J. (2018). Oral immunization with a Lactobacillus casei-based anti-porcine epidemic diarrhoea virus (PEDV) vaccine expressing microfold cell-targeting peptide Co1 fused with the COE antigen of PEDV. J. Appl. Microbiol..

[18] Bae J.L., Lee J.G., Kang T.J., Jang H.S., Jang Y.S., Yang M.S. (2003). Induction of antigen-specific systemic and mucosal immune responses by feeding animals transgenic plants expressing the antigen. Vaccine.

[19] Hofmann M., Wyler R. (1988). Propagation of the virus of porcine epidemic diarrhea in cell culture. J. Clin. Microbiol..

[20] Egberink H.F., Ederveen J., Callebaut P., Horzinek M.C. (1988). Characterization of the structural proteins of porcine epizootic diarrhea virus, strain CV777. Am. J. Vet. Res..

[21] Utiger A., Tobler K., Bridgen A., Ackermann M. (1995). Identification of the membrane protein of porcine epidemic diarrhea virus. Virus Genes.

[22] Myint S.H., Siddell S.G. (1995). Human coronavirus infections. The Coronaviridae.

[23] Drosten C., Gunther S., Preiser W., van der Werf S., Brodt H.R., Becker S., Rabenau H., Panning M., Kolesnikova L., Fouchier R.A. (2003). Identification of a novel coronavirus in patients with severe acute respiratory syndrome. N. Engl. J. Med..

[24] World Health Organization (2003). Global surveillance for severe acute respiratory syndrome (SARS). Wkly. Epidemiol. Rec..

[25] Zaki A.M., van Boheemen S., Bestebroer T.M., Osterhaus A.D., Fouchier R.A. (2012). Isolation of a novel coronavirus from a man with pneumonia in Saudi Arabia. N. Engl. J. Med..

[26] Azhar E.I., Hui D.S.C., Memish Z.A., Drosten C., Zumla A. (2019). The Middle East Respiratory Syndrome (MERS). Infect. Dis. Clin. North. Am..

[27] Hui D.S., Azhar E.I., Madani T.A., Ntoumi F., Kock R., Dar O., Ippolito G., McHugh T.D., Memish Z.A., Drosten C. (2020). The continuing 2019-nCoV epidemic threat of novel coronaviruses to global health—The latest 2019 novel coronavirus outbreak in Wuhan, China. Int. J. Infect. Dis..

[28] Wang W., Tang J., Wei F. (2020). Updated understanding of the outbreak of 2019 novel coronavirus (2019-nCoV) in Wuhan, China. J. Med. Virol..

[29] Pan Y., Tian X., Qin P., Wang B., Zhao P., Yang Y.L., Wang L., Wang D., Song Y., Zhang X. (2017). Discovery of a novel swine enteric alphacoronavirus (SeACoV) in southern China. Vet. Microbiol..

[30] Woo P.C., Lau S.K., Lam C.S., Lau C.C., Tsang A.K., Lau J.H., Bai R., Teng J.L., Tsang C.C., Wang M. (2012). Discovery of seven novel Mammalian and avian coronaviruses in the genus deltacoronavirus supports bat coronaviruses as the gene source of alphacoronavirus and betacoronavirus and avian coronaviruses as the gene source of gammacoronavirus and deltacoronavirus. J. Virol..

[31] Opriessnig T., Gimenez-Lirola L.G., Halbur P.G. (2011). Polymicrobial respiratory disease in pigs. Anim. Health Res. Rev..

[32] Li B.X., Ge J.W., Li Y.J. (2007). Porcine aminopeptidase N is a functional receptor for the PEDV coronavirus. Virology.

[33] Nam E., Lee C. (2010). Contribution of the porcine aminopeptidase N (CD13) receptor density to porcine epidemic diarrhea virus infection. Vet. Microbiol..

[34] Deng F., Ye G., Liu Q.Q., Navid M.T., Zhong X.L., Li Y.W., Wan C.Y., Xiao S.B., He Q.G., Fu Z.F. (2016). Identification and Comparison of Receptor Binding Characteristics of the Spike Protein of Two Porcine Epidemic Diarrhea Virus Strains. Viruses.

[35] Mina-Osorio P. (2008). The moonlighting enzyme CD13: Old and new functions to target. Trends Mol. Med..

[36] Liu C., Tang J., Ma Y., Liang X., Yang Y., Peng G., Qi Q., Jiang S., Li J., Du L. (2015). Receptor usage and cell entry of porcine epidemic diarrhea coronavirus. J. Virol..

[37] Gallagher T., Perlman S. (2013). Public health: Broad reception for coronavirus. Nature.

[38] Raj V.S., Mou H., Smits S.L., Dekkers D.H., Muller M.A., Dijkman R., Muth D., Demmers J.A., Zaki A., Fouchier R.A. (2013). Dipeptidyl peptidase 4 is a functional receptor for the emerging human coronavirus-EMC. Nature.

[39] Duarte M., Gelfi J., Lambert P., Rasschaert D., Laude H. (1993). Genome organization of porcine epidemic diarrhoea virus. Adv. Exp. Med. Biol..

[40] Song D., Park B. (2012). Porcine epidemic diarrhoea virus: A comprehensive review of molecular epidemiology, diagnosis, and vaccines. Virus Genes.

[41] Li R., Qiao S., Yang Y., Guo J., Xie S., Zhou E., Zhang G. (2016). Genome sequencing and analysis of a novel recombinant porcine epidemic diarrhea virus strain from Henan, China. Virus Genes.

[42] Subissi L., Imbert I., Ferron F., Collet A., Coutard B., Decroly E., Canard B. (2014). SARS-CoV ORF1b-encoded nonstructural proteins 12-16: Replicative enzymes as antiviral targets. Antivir. Res..

[43] Snijder E.J., Decroly E., Ziebuhr J. (2016). The Nonstructural Proteins Directing Coronavirus RNA Synthesis and Processing. Adv. Virus Res..

[44] Xu X., Zhang H., Zhang Q., Huang Y., Dong J., Liang Y., Liu H.J., Tong D. (2013). Porcine epidemic diarrhea virus N protein prolongs S-phase cell cycle, induces endoplasmic reticulum stress, and up-regulates interleukin-8 expression. Vet. Microbiol..

[45] Shirato K., Matsuyama S., Ujike M., Taguchi F. (2011). Role of proteases in the release of porcine epidemic diarrhea virus from infected cells. J. Virol..

[46] Li C., Li Z., Zou Y., Wicht O., Kuppeveld F.J.M.V., Rottier P.J.M., Bosch B.J. (2013). Manipulation of the Porcine Epidemic Diarrhea Virus Genome Using Targeted RNA Recombination. PLoS ONE.

[47] van Hemert M.J., van den Worm S.H.E., Knoops K., Mommaas A.M., Gorbalenya A.E., Snijder E.J. (2008). SARS-Coronavirus Replication/Transcription Complexes Are Membrane-Protected and Need a Host Factor for Activity In Vitro. PLoS Pathog..

[48] Li F. (2016). Structure, Function, and Evolution of Coronavirus Spike Proteins. Annu. Rev. Virol..

[49] Hulswit R.J., de Haan C.A., Bosch B.J. (2016). Coronavirus Spike Protein and Tropism Changes. Adv. Virus Res..

[50] Walls A.C., Tortorici M.A., Bosch B.J., Frenz B., Rottier P.J.M., DiMaio F., Rey F.A., Veesler D. (2016). Cryo-electron microscopy structure of a coronavirus spike glycoprotein trimer. Nature.

[51] Tresnan D.B., Levis R., Holmes K.V. (1996). Feline aminopeptidase N serves as a receptor for feline, canine, porcine, and human coronaviruses in serogroup I. J. Virol..

[52] Delmas B., Gelfi J., L’Haridon R., Vogel L.K., Sjöström H., Norén O., Laude H. (1992). Aminopeptidase N is a major receptor for the enteropathogenic coronavirus TGEV. Nature.

[53] Schultze B., Krempl C., Ballesteros M.L., Shaw L., Schauer R., Enjuanes L., Herrler G. (1996). Transmissible gastroenteritis coronavirus, but not the related porcine respiratory coronavirus, has a sialic acid (N-glycolylneuraminic acid) binding activity. J. Virol..

[54] Yeager C.L., Ashmun R.A., Williams R.K., Cardellichio C.B., Shapiro L.H., Look A.T., Holmes K.V. (1992). Human aminopeptidase N is a receptor for human coronavirus 229E. Nature.

[55] Li Z., Chen F., Yuan Y., Zeng X., Wei Z., Zhu L., Sun B., Xie Q., Cao Y., Xue C. (2013). Sequence and phylogenetic analysis of nucleocapsid genes of porcine epidemic diarrhea virus (PEDV) strains in China. Arch. Virol..

[56] Tan Y.W., Fang S., Fan H., Lescar J., Liu D.X. (2006). Amino acid residues critical for RNA-binding in the N-terminal domain of the nucleocapsid protein are essential determinants for the infectivity of coronavirus in cultured cells. Nucleic Acids Res..

[57] Fan H., Ooi A., Tan Y.W., Wang S., Fang S., Liu D.X., Lescar J. (2005). The nucleocapsid protein of coronavirus infectious bronchitis virus: Crystal structure of its N-terminal domain and multimerization properties. Structure.

[58] Ding Z., Fang L., Jing H., Zeng S., Wang D., Liu L., Zhang H., Luo R., Chen H., Xiao S. (2014). Porcine epidemic diarrhea virus nucleocapsid protein antagonizes beta interferon production by sequestering the interaction between IRF3 and TBK1. J. Virol..

[59] Lu X., Pan J., Tao J., Guo D. (2011). SARS-CoV nucleocapsid protein antagonizes IFN-β response by targeting initial step of IFN-β induction pathway, and its C-terminal region is critical for the antagonism. Virus Genes.

[60] Ye Y., Hauns K., Langland J.O., Jacobs B.L., Hogue B.G. (2007). Mouse hepatitis coronavirus A59 nucleocapsid protein is a type I interferon antagonist. J. Virol..

[61] Ding Z., Fang L., Yuan S., Zhao L., Wang X., Long S., Wang M., Wang D., Foda M.F., Xiao S. (2017). The nucleocapsid proteins of mouse hepatitis virus and severe acute respiratory syndrome coronavirus share the same IFN-β antagonizing mechanism: Attenuation of PACT-mediated RIG-I/MDA5 activation. Oncotarget.

[62] Pulford D.J., Britton P. (1991). Expression and cellular localisation of porcine transmissible gastroenteritis virus N and M proteins by recombinant vaccinia viruses. Virus Res..

[63] De Haan C.A., Kuo L., Masters P.S., Vennema H., Rottier P.J. (1998). Coronavirus particle assembly: Primary structure requirements of the membrane protein. J. Virol..

[64] Xu X.G., Zhang H.L., Zhang Q., Dong J., Huang Y., Tong D.W. (2015). Porcine epidemic diarrhea virus M protein blocks cell cycle progression at S-phase and its subcellular localization in the porcine intestinal epithelial cells. Acta Virol..

[65] Zhang Q., Shi K., Yoo D. (2016). Suppression of type I interferon production by porcine epidemic diarrhea virus and degradation of CREB-binding protein by nsp1. Virology.

[66] Brian D.A., Baric R.S. (2005). Coronavirus genome structure and replication. Curr. Top. Microbiol. Immunol..

[67] Xu X., Zhang H., Zhang Q., Dong J., Liang Y., Huang Y., Liu H.J., Tong D. (2013). Porcine epidemic diarrhea virus E protein causes endoplasmic reticulum stress and up-regulates interleukin-8 expression. Virol. J..

[68] Wang K., Lu W., Chen J., Xie S., Shi H., Hsu H., Yu W., Xu K., Bian C., Fischer W.B. (2012). PEDV ORF3 encodes an ion channel protein and regulates virus production. FEBS Lett..

[69] Ye S., Li Z., Chen F., Li W., He Q. (2015). Porcine epidemic diarrhea virus ORF3 gene prolongs S-phase, facilitates formation of vesicles and promotes the proliferation of attenuated PEDV. Virus Genes.

[70] Challika K., Qigai H., Anan J. (2008). The Accessory Protein ORF3 Contributes to Porcine Epidemic Diarrhea Virus Replication by Direct Binding to the Spike Protein. Viruses.

[71] Ziebuhr J. (2005). The coronavirus replicase. Curr. Top. Microbiol. Immunol..

[72] Woo P.C.Y., Huang Y., Lau S.K.P., Yuen K.-Y. (2010). Coronavirus Genomics and Bioinformatics Analysis. Viruses.

[73] Connor R.F., Roper R.L. (2007). Unique SARS-CoV protein nsp1: Bioinformatics, biochemistry and potential effects on virulence. Trends Microbiol..

[74] Jansson A.M. (2013). Structure of alphacoronavirus transmissible gastroenteritis virus nsp1 has implications for coronavirus nsp1 function and evolution. J. Virol..

[75] Snijder E.J., Bredenbeek P.J., Dobbe J.C., Thiel V., Ziebuhr J., Poon L.L., Guan Y., Rozanov M., Spaan W.J., Gorbalenya A.E. (2003). Unique and conserved features of genome and proteome of SARS-coronavirus, an early split-off from the coronavirus group 2 lineage. J. Mol. Biol..

[76] Narayanan K., Ramirez S.I., Lokugamage K.G., Makino S. (2015). Coronavirus nonstructural protein 1: Common and distinct functions in the regulation of host and viral gene expression. Virus Res..

[77] Shen Z., Wang G., Yang Y., Shi J., Peng G. (2019). A conserved region of nonstructural protein 1 from alphacoronaviruses inhibits host gene expression and is critical for viral virulence. J. Biol. Chem..

[78] Zhang Q., Ma J., Yoo D. (2017). Inhibition of NF-κB activity by the porcine epidemic diarrhea virus nonstructural protein 1 for innate immune evasion. Virology.

[79] Graham R.L., Sims A.C., Brockway S.M., Baric R.S., Denison M.R. (2005). The nsp2 replicase proteins of murine hepatitis virus and severe acute respiratory syndrome coronavirus are dispensable for viral replication. J. Virol..

[80] Xing Y., Chen J., Tu J., Zhang B., Chen X., Shi H., Baker S.C., Feng L., Chen Z. (2013). The papain-like protease of porcine epidemic diarrhea virus negatively regulates type I interferon pathway by acting as a viral deubiquitinase. J. Gen. Virol..

[81] Clementz M.A., Chen Z., Banach B.S., Wang Y., Sun L., Ratia K., Baez-Santos Y.M., Wang J., Takayama J., Ghosh A.K. (2010). Deubiquitinating and Interferon Antagonism Activities of Coronavirus Papain-Like Proteases. J. Virol..

[82] Miller S., Krijnse-Locker J. (2008). Modification of intracellular membrane structures for virus replication. Nat. Rev. Microbiol..

[83] Salonen A., Ahola T., Kääriäinen L. (2005). Viral RNA Replication in Association with Cellular Membranes. Curr. Top. Microbiol. Immunol..

[84] Knoops K., Kikkert M., van den Worm S.H.E., Zevenhoven-Dobbe J.C., van der Meer Y., Koster A.J., Mommaas A.M., Snijder E.J., Emerman M. (2008). SARS-Coronavirus Replication Is Supported by a Reticulovesicular Network of Modified Endoplasmic Reticulum. PLOS Biol..

[85] Gosert R., Kanjanahaluethai A., Egger D., Bienz K., Baker S.C. (2002). RNA Replication of Mouse Hepatitis Virus Takes Place at Double-Membrane Vesicles. J. Virol..

[86] De Wilde A.H., Raj V.S., Oudshoorn D., Bestebroer T.M., van Nieuwkoop S., Limpens R.W.A.L., Posthuma C.C., van der Meer Y., Barcena M., Haagmans B.L. (2002). MERS-coronavirus replication induces severe in vitro cytopathology and is strongly inhibited by cyclosporin A or interferon-α treatment. J. Gen. Virol..

[87] Snijder E.J., van der Meer Y., Zevenhoven-Dobbe J., Onderwater J.J.M., van der Meulen J., Koerten H.K., Mommaas A.M. (2006). Ultrastructure and Origin of Membrane Vesicles Associated with the Severe Acute Respiratory Syndrome Coronavirus Replication Complex. J. Virol..

[88] Goldsmith C.S., Tatti K.M., Ksiazek T.G., Rollin P.E., Comer J.A., Lee W.W., Rota P.A., Bankamp B., Bellini W.J., Zaki S.R. (2004). Ultrastructural Characterization of SARS Coronavirus. Emerg. Infect. Dis..

[89] Ulasli M., Verheije M.H., Haan C.A.M.d., Reggiori F. (2010). Qualitative and quantitative ultrastructural analysis of the membrane rearrangements induced by coronavirus. Cell Microbiol..

[90] Orenstein J.M., Banach B.S., Baker S.C. (2008). Morphogenesis of Coronavirus HCoV-NL63 in Cell Culture: A Transmission Electron Microscopic Study. Open Infect. Dis. J..

[91] Lundin A., Dijkman R., Bergström T., Kann N., Adamiak B., Hannoun C., Kindler E., Jónsdóttir H.R., Muth D., Kint J. (2004). Targeting Membrane-Bound Viral RNA Synthesis Reveals Potent Inhibition of Diverse Coronaviruses Including the Middle East Respiratory Syndrome Virus. PLoS Pathog..

[92] Ávila-Pérez G., Rejas M.T., Rodríguez D. (2006). Ultrastructural characterization of membranous torovirus replication factories. Cell. Microbiol..

[93] Reggiori F., Monastyrska I., Verheije M.H., Cali T., Ulasli M., Bianchi S., Bernasconi R., de Haan C.A., Molinari M. (2010). Coronaviruses Hijack the LC3-I-positive EDEMosomes, ER-derived vesicles exporting short-lived ERAD regulators, for replication. Cell Host Microbe.

[94] Sawicki S.G., Sawicki D.L., Siddell S.G. (2007). A contemporary view of coronavirus transcription. J. Virol..

[95] Oudshoorn D., Rijs K., Limpens R., Groen K., Koster A.J., Snijder E.J., Kikkert M., Barcena M. (2017). Expression and Cleavage of Middle East Respiratory Syndrome Coronavirus nsp3-4 Polyprotein Induce the Formation of Double-Membrane Vesicles That Mimic Those Associated with Coronaviral RNA Replication. mBio.

[96] Angelini M.M., Akhlaghpour M., Neuman B.W., Buchmeier M.J. (2013). Severe acute respiratory syndrome coronavirus nonstructural proteins 3, 4, and 6 induce double-membrane vesicles. mBio.

[97] Wojdyla J.A., Manolaridis I., van Kasteren P.B., Kikkert M., Snijder E.J., Gorbalenya A.E., Tucker P.A. (2010). Papain-like protease 1 from transmissible gastroenteritis virus: Crystal structure and enzymatic activity toward viral and cellular substrates. J. Virol..

[98] Sawicki S.G., Sawicki D.L., Younker D., Meyer Y., Thiel V., Stokes H., Siddell S.G. (2005). Functional and genetic analysis of coronavirus replicase-transcriptase proteins. PLoS Pathog..

[99] Perlman S., Netland J. (2009). Coronaviruses post-SARS: Update on replication and pathogenesis. Nat. Rev. Microbiol..

[100] Wang D., Fang L., Shi Y., Zhang H., Gao L., Peng G., Chen H., Li K., Xiao S. (2015). Porcine Epidemic Diarrhea Virus 3C-Like Protease Regulates Its Interferon Antagonism by Cleaving NEMO. J. Virol..

[101] Anand K., Palm G.J., Mesters J.R., Siddell S.G., Ziebuhr J., Hilgenfeld R. (2002). Structure of coronavirus main proteinase reveals combination of a chymotrypsin fold with an extra alpha-helical domain. EMBO J..

[102] Peti W., Johnson M.A., Herrmann T., Neuman B.W., Buchmeier M.J., Nelson M., Joseph J., Page R., Stevens R.C., Kuhn P. (2005). Structural genomics of the severe acute respiratory syndrome coronavirus: Nuclear magnetic resonance structure of the protein nsP7. J. Virol..

[103] Ratia K., Saikatendu K.S., Santarsiero B.D., Barretto N., Baker S.C., Stevens R.C., Mesecar A.D. (2006). Severe acute respiratory syndrome coronavirus papain-like protease: Structure of a viral deubiquitinating enzyme. Proc. Natl. Acad. Sci. USA.

[104] Saikatendu K.S., Joseph J.S., Subramanian V., Clayton T., Griffith M., Moy K., Velasquez J., Neuman B.W., Buchmeier M.J., Stevens R.C. (2005). Structural basis of severe acute respiratory syndrome coronavirus ADP-ribose-1″-phosphate dephosphorylation by a conserved domain of nsP3. Structure.

[105] Sutton G., Fry E., Carter L., Sainsbury S., Walter T., Nettleship J., Berrow N., Owens R., Gilbert R., Davidson A. (2004). The nsp9 replicase protein of SARS-coronavirus, structure and functional insights. Structure.

[106] Zhai Y., Sun F., Li X., Pang H., Xu X., Bartlam M., Rao Z. (2005). Insights into SARS-CoV transcription and replication from the structure of the nsp7-nsp8 hexadecamer. Nat. Struct. Mol. Biol..

[107] Joseph J.S., Saikatendu K.S., Subramanian V., Neuman B.W., Brooun A., Griffith M., Moy K., Yadav M.K., Velasquez J., Buchmeier M.J. (2006). Crystal structure of nonstructural protein 10 from the severe acute respiratory syndrome coronavirus reveals a novel fold with two zinc-binding motifs. J. Virol..

[108] Egloff M.-P., Ferron F., Campanacci V., Longhi S., Rancurel C., Dutartre H., Snijder E.J., Gorbalenya A.E., Cambillau C., Canard B. (2004). The severe acute respiratory syndrome-coronavirus replicative protein nsp9 is a single-stranded RNA-binding subunit unique in the RNA virus world. Proc. Natl. Acad. Sci. USA.

[109] Kirchdoerfer R.N., Ward A.B. (2019). Structure of the SARS-CoV nsp12 polymerase bound to nsp7 and nsp8 co-factors. Nat. Commun..

[110] Subissi L., Posthuma C.C., Collet A., Zevenhoven-Dobbe J.C., Gorbalenya A.E., Decroly E., Snijder E.J., Canard B., Imbert I. (2014). One severe acute respiratory syndrome coronavirus protein complex integrates processive RNA polymerase and exonuclease activities. Proc. Natl. Acad. Sci. USA.

[111] Fang S., Chen B., Tay F.P.L., Ng B.S., Liu D.X. (2007). An arginine-to-proline mutation in a domain with undefined functions within the helicase protein (Nsp13) is lethal to the coronavirus infectious bronchitis virus in cultured cells. Virology.

[112] Chen Y., Cai H., Pan J., Xiang N., Tien P., Ahola T., Guo D. (2009). Functional screen reveals SARS coronavirus nonstructural protein nsp14 as a novel cap N7 methyltransferase. Proc. Natl. Acad. Sci. USA.

[113] Minskaia E., Hertzig T., Gorbalenya A.E., Campanacci V., Cambillau C., Canard B., Ziebuhr J. (2006). Discovery of an RNA virus 3′->5′ exoribonuclease that is critically involved in coronavirus RNA synthesis. Proc. Natl. Acad. Sci. USA.

[114] Cao J., Wu C.C., Lin T.L. (2008). Turkey coronavirus non-structure protein NSP15--an endoribonuclease. Intervirology.

[115] Xu X., Zhai Y., Sun F., Lou Z., Su D., Xu Y., Zhang R., Joachimiak A., Zhang X.C., Bartlam M. (2006). New antiviral target revealed by the hexameric structure of mouse hepatitis virus nonstructural protein nsp15. J. Virol..

[116] Guarino L.A., Bhardwaj K., Dong W., Sun J., Holzenburg A., Kao C. (2005). Mutational analysis of the SARS virus Nsp15 endoribonuclease: Identification of residues affecting hexamer formation. J. Mol. Biol..

[117] Kindler E., Gil-Cruz C., Spanier J., Li Y., Wilhelm J., Rabouw H.H., Zust R., Hwang M., V’Kovski P., Stalder H. (2017). Early endonuclease-mediated evasion of RNA sensing ensures efficient coronavirus replication. PLoS Pathog..

[118] Deng X., Hackbart M., Mettelman R.C., O’Brien A., Mielech A.M., Yi G., Kao C.C., Baker S.C. (2017). Coronavirus nonstructural protein 15 mediates evasion of dsRNA sensors and limits apoptosis in macrophages. Proc. Natl. Acad. Sci. USA.

[119] Decroly E., Ferron F., Lescar J., Canard B. (2012). Conventional and unconventional mechanisms for capping viral mRNA. Nat. Rev. Microbiol..

[120] Hyde J.L., Diamond M.S. (2015). Innate immune restriction and antagonism of viral RNA lacking 2-O methylation. Virology.

[121] Zust R., Cervantes-Barragan L., Habjan M., Maier R., Neuman B.W., Ziebuhr J., Szretter K.J., Baker S.C., Barchet W., Diamond M.S. (2011). Ribose 2′-O-methylation provides a molecular signature for the distinction of self and non-self mRNA dependent on the RNA sensor Mda5. Nat. Immunol..

[122] Ivanov K.A., Thiel V., Dobbe J.C., van der Meer Y., Snijder E.J., Ziebuhr J. (2004). Multiple enzymatic activities associated with severe acute respiratory syndrome coronavirus helicase. J. Virol..

[123] Decroly E., Imbert I., Coutard B., Bouvet M., Selisko B., Alvarez K., Gorbalenya A.E., Snijder E.J., Canard B. (2008). Coronavirus Nonstructural Protein 16 Is a Cap-0 Binding Enzyme Possessing (Nucleoside-2′*O*)-Methyltransferase Activity. J. Virol..

[124] Chen Y., Su C., Ke M., Jin X., Xu L., Zhang Z., Wu A., Sun Y., Yang Z., Tien P. (2011). Biochemical and Structural Insights into the Mechanisms of SARS Coronavirus RNA Ribose 2′-O-Methylation by nsp16/nsp10 Protein Complex. PLoS Pathog..

[125] Ma Y., Wu L., Shaw N., Gao Y., Wang J., Sun Y., Lou Z., Yan L., Zhang R., Rao Z. (2015). Structural basis and functional analysis of the SARS coronavirus nsp14-nsp10 complex. Proc. Natl. Acad. Sci. USA.

[126] Bouvet M., Lugari A., Posthuma C.C., Zevenhoven J.C., Bernard S., Betzi S., Imbert I., Canard B., Guillemot J.C., Lecine P. (2014). Coronavirus Nsp10, a critical co-factor for activation of multiple replicative enzymes. J. Biol. Chem..

[127] Thiel V., Ivanov K.A., Putics A., Hertzig T., Schelle B., Bayer S., Weissbrich B., Snijder E.J., Rabenau H., Doerr H.W. (2003). Mechanisms and enzymes involved in SARS coronavirus genome expression. J. Gen. Virol..

[128] Chen Y., Zhang Z., Li J., Gao Y., Zhou L., Ge X., Han J., Guo X., Yang H. (2018). Porcine epidemic diarrhea virus S1 protein is the critical inducer of apoptosis. Virol. J..

[129] Shi D., Shi H., Sun D., Chen J., Zhang X., Wang X., Zhang J., Ji Z., Liu J., Cao L. (2017). Nucleocapsid Interacts with NPM1 and Protects it from Proteolytic Cleavage, Enhancing Cell Survival, and is Involved in PEDV Growth. Sci. Rep..

[130] Zhang Q., Ke H., Blikslager A., Fujita T., Yoo D. (2018). Type III Interferon Restriction by Porcine Epidemic Diarrhea Virus and the Role of Viral Protein nsp1 in IRF1 Signaling. J. Virol..

[131] Zeng Z., Deng F., Shi K., Ye G., Wang G., Fang L., Xiao S., Fu Z., Peng G. (2018). Dimerization of coronavirus nsp9 with diverse modes enhances its nucleic acid binding affinity. J. Virol..

[132] Shi P., Su Y., Li R., Liang Z., Dong S., Huang J. (2019). PEDV nsp16 negatively regulates innate immunity to promote viral proliferation. Virus Res..

[133] Li H.-J., Wang X.-X., Gao D.-S., Huang H.-M., Chen L., Chang H.-T., Wang C.-Q., Li Y.-T., Zhao J. (2017). Subcellular Localization and Effect on Type I Interferon Response of Porcine Epidemic Diarrhea Virus Nsp7. Chin. J. Anim. Vet. Sci..

[134] De Wilde A.H., Snijder E.J., Kikkert M., van Hemert M.J. (2018). Host Factors in Coronavirus Replication. Curr. Top. Microbiol. Immunol..

[135] Deng X., van Geelen A., Buckley A.C., O’Brien A., Pillatzki A., Lager K.M., Faaberg K.S., Baker S.C. (2019). Coronavirus Endoribonuclease Activity in Porcine Epidemic Diarrhea Virus Suppresses Type I and Type III Interferon Responses. J. Virol..

[136] Takeuchi O., Akira S. (2009). Innate immunity to virus infection. Immunol. Rev..

[137] Yoneyama M., Onomoto K., Jogi M., Akaboshi T., Fujita T. (2015). Viral RNA detection by RIG-I-like receptors. Curr. Opin. Immunol..

[138] Kawai T., Akira S. (2008). Toll-like receptor and RIG-I-like receptor signaling. Ann. N. Y. Acad. Sci..

[139] Sparrer K.M., Gack M.U. (2015). Intracellular detection of viral nucleic acids. Curr. Opin. Microbiol..

[140] Fujita T. (2009). A nonself RNA pattern: Tri-p to panhandle. Immunity.

[141] Yoneyama M., Kikuchi M., Natsukawa T., Shinobu N., Imaizumi T., Miyagishi M., Taira K., Akira S., Fujita T. (2004). The RNA helicase RIG-I has an essential function in double-stranded RNA-induced innate antiviral responses. Nat. Immunol..

[142] Durbin R.K., Kotenko S.V., Durbin J.E. (2013). Interferon induction and function at the mucosal surface. Immunol. Rev..

[143] Zhu Z., Zhang X., Wang G., Zheng H. (2014). The laboratory of genetics and physiology 2: Emerging insights into the controversial functions of this RIG-I-like receptor. Biomed. Res. Int..

[144] Satoh T., Kato H., Kumagai Y., Yoneyama M., Sato S., Matsushita K., Tsujimura T., Fujita T., Akira S., Takeuchi O. (2010). LGP2 is a positive regulator of RIG-I- and MDA5-mediated antiviral responses. Proc. Natl. Acad. Sci. USA.

[145] Rothenfusser S., Goutagny N., DiPerna G., Gong M., Monks B.G., Schoenemeyer A., Yamamoto M., Akira S., Fitzgerald K.A. (2005). The RNA helicase Lgp2 inhibits TLR-independent sensing of viral replication by retinoic acid-inducible gene-I. J. Immunol..

[146] Jin H.S., Suh H.W., Kim S.J., Jo E.K. (2017). Mitochondrial Control of Innate Immunity and Inflammation. Immune. Netw..

[147] Sun L., Liu S., Chen Z.J. (2010). SnapShot: Pathways of antiviral innate immunity. Cell.

[148] Zhang Q., Yoo D. (2016). Immune evasion of porcine enteric coronaviruses and viral modulation of antiviral innate signaling. Virus Res..

[149] Fang R., Jiang Q., Zhou X., Wang C., Guan Y., Tao J., Xi J., Feng J.M., Jiang Z. (2017). MAVS activates TBK1 and IKKε through TRAFs in NEMO dependent and independent manner. PLoS Pathog..

[150] Bhoj V.G., Chen Z.J. (2009). Ubiquitylation in innate and adaptive immunity. Nature.

[151] Kowalinski E., Lunardi T., McCarthy A.A., Louber J., Brunel J., Grigorov B., Gerlier D., Cusack S. (2011). Structural basis for the activation of innate immune pattern-recognition receptor RIG-I by viral RNA. Cell.

[152] Jiang F., Ramanathan A., Miller M.T., Tang G.Q., Gale M., Patel S.S., Marcotrigiano J. (2011). Structural basis of RNA recognition and activation by innate immune receptor RIG-I. Nature.

[153] Luo D., Ding S.C., Vela A., Kohlway A., Lindenbach B.D., Pyle A.M. (2011). Structural insights into RNA recognition by RIG-I. Cell.

[154] Brautigam C.A. (2012). Ubiquitin-induced oligomerization of the RNA sensors RIG-I and MDA5 activates antiviral innate immune response. Immunity.

[155] Zeng W., Sun L., Jiang X., Chen X., Hou F., Adhikari A., Xu M., Chen Z.J. (2010). Reconstitution of the RIG-I Pathway Reveals a Pivotal Role of Unanchored Polyubiquitin Chains in Innate Immunity. Cell.

[156] Sun S.C. (2008). Deubiquitylation and regulation of the immune response. Nat. Rev. Immunol..

[157] Stark G.R., Darnell J.E. (2012). The JAK-STAT pathway at twenty. Immunity.

[158] Okamura H., Kashiwamura S., Tsutsui H., Yoshimoto T., Nakanishi K. (1998). Regulation of interferon-gamma production by IL-12 and IL-18. Curr. Opin. Immunol..

[159] Carlin A.F., Plummer E.M., Vizcarra E.A., Sheets N., Joo Y., Tang W., Day J., Greenbaum J., Glass C.K., Diamond M.S. (2017). An IRF-3-, IRF-5-, and IRF-7-Independent Pathway of Dengue Viral Resistance Utilizes IRF-1 to Stimulate Type I and II Interferon Responses. Cell Rep..

[160] Hermant P., Michiels T. (2014). Interferon-λ in the context of viral infections: Production, response and therapeutic implications. J. Innate Immun..

[161] Kotenko S.V., Gallagher G., Baurin V.V., Lewis-Antes A., Donnelly R.P. (2003). IFN-λs mediate antiviral protection through a distinct class II cytokine receptor complex. Nat. Immunol..

[162] Prokunina-Olsson L., Muchmore B., Tang W., Pfeiffer R.M., Park H., Dickensheets H., Hergott D., Porter-Gill P., Mumy A., Kohaar I. (2013). A variant upstream of IFNL3 (IL28B) creating a new interferon gene IFNL4 is associated with impaired clearance of hepatitis C virus. Nat. Genet..

[163] Kumar H., Kawai T., Akira S. (2009). Toll-like receptors and innate immunity. Biochem. Biophys. Res. Commun..

[164] Thomson S.J., Goh F.G., Banks H., Krausgruber T., Kotenko S.V., Foxwell B.M., Udalova I.A. (2009). The role of transposable elements in the regulation of IFN-λ1 gene expression. Proc. Natl. Acad. Sci. USA.

[165] Sheppard P., Kindsvogel W., Xu W., Henderson K., Schlutsmeyer S., Whitmore T.E., Kuestner R., Garrigues U., Birks C., Roraback J. (2003). IL-28, IL-29 and their class II cytokine receptor IL-28R. Nat. Immunol..

[166] Kotenko S.V., Rivera A., Parker D., Durbin J.E. (2019). Type III IFNs: Beyond antiviral protection. Semin. Immunol..

[167] Kindler E., Thiel V. (2014). To sense or not to sense viral RNA--essentials of coronavirus innate immune evasion. Curr. Opin. Microbiol..

[168] Shan Y., Liu Z.Q., Li G.W., Chen C., Luo H., Liu Y.J., Zhuo X.H., Shi X.F., Fang W.H., Li X.L. (2018). Nucleocapsid protein from porcine epidemic diarrhea virus isolates can antagonize interferon-λ production by blocking the nuclear factor-κB nuclear translocation. J. Zhejiang Univ. Sci. B (Biomed. Biotechnol.).

[169] Cao L., Ge X., Gao Y., Herrler G., Ren Y., Ren X., Li G. (2015). Porcine epidemic diarrhea virus inhibits dsRNA-induced interferon-β production in porcine intestinal epithelial cells by blockade of the RIG-I-mediated pathway. Virol. J..

[170] Guo L., Luo X., Li R., Xu Y., Zhang J., Ge J., Bu Z., Feng L., Wang Y. (2016). Porcine Epidemic Diarrhea Virus Infection Inhibits Interferon Signaling by Targeted Degradation of STAT1. J. Virol..

[171] Hu Y., Li W., Gao T., Cui Y., Jin Y., Li P., Ma Q., Liu X., Cao C. (2017). The Severe Acute Respiratory Syndrome Coronavirus Nucleocapsid Inhibits Type I Interferon Production by Interfering with TRIM25-Mediated RIG-I Ubiquitination. J. Virol..

[172] Luo C., Luo H., Zheng S., Gui C., Yue L., Yu C., Sun T., He P., Chen J., Shen J. (2004). Nucleocapsid protein of SARS coronavirus tightly binds to human cyclophilin A. Biochem. Biophys. Res. Commun..

[173] Wang Q., Li C., Zhang Q., Wang T., Li J., Guan W., Yu J., Liang M., Li D. (2010). Interactions of SARS coronavirus nucleocapsid protein with the host cell proteasome subunit p42. Virol. J..

[174] Zhao X., Nicholls J.M., Chen Y.G. (2008). Severe acute respiratory syndrome-associated coronavirus nucleocapsid protein interacts with Smad3 and modulates transforming growth factor-β signaling. J. Biol. Chem..

[175] Wang Y., Zhang X. (1999). The nucleocapsid protein of coronavirus mouse hepatitis virus interacts with the cellular heterogeneous nuclear ribonucleoprotein A1 in vitro and in vivo. Virology.

[176] Zhang Y.P., Zhang R.W., Chang W.S., Wang Y.Y. (2010). Cxcl16 interact with SARS-CoV N protein in and out cell. Virol. Sin..

[177] Ziebuhr J., Schelle B., Karl N., Minskaia E., Bayer S., Siddell S.G., Gorbalenya A.E., Thiel V. (2007). Human coronavirus 229E papain-like proteases have overlapping specificities but distinct functions in viral replication. J. Virol..

[178] Almeida M.S., Johnson M.A., Herrmann T., Geralt M., Wuthrich K. (2007). Novel beta-barrel fold in the nuclear magnetic resonance structure of the replicase nonstructural protein 1 from the severe acute respiratory syndrome coronavirus. J. Virol..

[179] Narayanan K., Huang C., Lokugamage K., Kamitani W., Ikegami T., Tseng C.T., Makino S. (2008). Severe acute respiratory syndrome coronavirus nsp1 suppresses host gene expression, including that of type I interferon, in infected cells. J. Virol..

[180] Huang C., Lokugamage K.G., Rozovics J.M., Narayanan K., Semler B.L., Makino S. (2011). SARS coronavirus nsp1 protein induces template-dependent endonucleolytic cleavage of mRNAs: Viral mRNAs are resistant to nsp1-induced RNA cleavage. PLoS Pathog..

[181] Kamitani W., Narayanan K., Huang C., Lokugamage K., Ikegami T., Ito N., Kubo H., Makino S. (2006). Severe acute respiratory syndrome coronavirus nsp1 protein suppresses host gene expression by promoting host mRNA degradation. Proc. Natl. Acad. Sci. USA.

[182] Wathelet M.G., Orr M., Frieman M.B., Baric R.S. (2007). Severe acute respiratory syndrome coronavirus evades antiviral signaling: Role of nsp1 and rational design of an attenuated strain. J. Virol..

[183] Shen Z., Ye G., Deng F., Wang G., Cui M., Fang L., Xiao S., Fu Z.F., Peng G. (2018). Structural Basis for the Inhibition of Host Gene Expression by Porcine Epidemic Diarrhea Virus nsp1. J. Virol..

[184] Dragan A.I., Hargreaves V.V., Makeyeva E.N., Privalov P.L. (2007). Mechanisms of activation of interferon regulator factor 3: The role of C-terminal domain phosphorylation in IRF-3 dimerization and DNA binding. Nucleic Acids Res..

[185] Lin R., Heylbroeck C., Pitha P.M., Hiscott J. (1998). Virus-dependent phosphorylation of the IRF-3 transcription factor regulates nuclear translocation, transactivation potential, and proteasome-mediated degradation. Mol. Cell. Biol..

[186] Panne D., Maniatis T., Harrison S.C. (2007). An atomic model of the interferon-β enhanceosome. Cell.

[187] Kumari P., Kumar H. (2017). Viral deubiquitinases: Role in evasion of anti-viral innate immunity. Crit. Rev. Microbiol..

[188] Hu H., Sun S.C. (2016). Ubiquitin signaling in immune responses. Cell Res..

[189] Harhaj E.W., Dixit V.M. (2011). Deubiquitinases in the regulation of NF-kappaB signaling. Cell Res..

[190] Zhang L., Zhao X., Zhang M., Zhao W., Gao C. (2014). Ubiquitin-specific protease 2b negatively regulates IFN-β production and antiviral activity by targeting TANK-binding kinase 1. J. Immunol..

[191] Wei R., Liu X., Yu W., Yang T., Cai W., Liu J., Huang X., Xu G.T., Zhao S., Yang J. (2015). Deubiquitinases in cancer. Oncotarget.

[192] Wang D., Fang L., Li P., Sun L., Fan J., Zhang Q., Luo R., Liu X., Li K., Chen H. (2011). The leader proteinase of foot-and-mouth disease virus negatively regulates the type I interferon pathway by acting as a viral deubiquitinase. J. Virol..

[193] Van Kasteren P.B., Beugeling C., Ninaber D.K., Frias-Staheli N., van Boheemen S., Garcia-Sastre A., Snijder E.J., Kikkert M. (2012). Arterivirus and nairovirus ovarian tumor domain-containing Deubiquitinases target activated RIG-I to control innate immune signaling. J. Virol..

[194] Sun Z., Chen Z., Lawson S.R., Fang Y. (2010). The cysteine protease domain of porcine reproductive and respiratory syndrome virus nonstructural protein 2 possesses deubiquitinating and interferon antagonism functions. J. Virol..

[195] Zheng D., Chen G., Guo B., Cheng G., Tang H. (2008). PLP2, a potent deubiquitinase from murine hepatitis virus, strongly inhibits cellular type I interferon production. Cell Res..

[196] Chen X., Yang X., Zheng Y., Yang Y., Xing Y., Chen Z. (2014). SARS coronavirus papain-like protease inhibits the type I interferon signaling pathway through interaction with the STING-TRAF3-TBK1 complex. Protein Cell.

[197] Van der Hoek L., Sure K., Ihorst G., Stang A., Pyrc K., Jebbink M.F., Petersen G., Forster J., Berkhout B., Uberla K. (2005). Croup is associated with the novel coronavirus NL63. PLoS Med..

[198] Yuan L., Chen Z., Song S., Wang S., Tian C., Xing G., Chen X., Xiao Z.X., He F., Zhang L. (2015). p53 degradation by a coronavirus papain-like protease suppresses type I interferon signaling. J. Biol. Chem..

[199] Wang D., Fang L., Li K., Zhong H., Fan J., Ouyang C., Zhang H., Duan E., Luo R., Zhang Z. (2012). Foot-and-mouth disease virus 3C protease cleaves NEMO to impair innate immune signaling. J. Virol..

[200] Wang D., Fang L., Wei D., Zhang H., Luo R., Chen H., Li K., Xiao S. (2014). Hepatitis A virus 3C protease cleaves NEMO to impair induction of beta interferon. J. Virol..

[201] Lei X., Han N., Xiao X., Jin Q., He B., Wang J. (2014). Enterovirus 71 3C inhibits cytokine expression through cleavage of the TAK1/TAB1/TAB2/TAB3 complex. J. Virol..

[202] Lei X., Sun Z., Liu X., Jin Q., He B., Wang J. (2011). Cleavage of the adaptor protein TRIF by enterovirus 71 3C inhibits antiviral responses mediated by Toll-like receptor 3. J. Virol..

[203] Mukherjee A., Morosky S.A., Delorme-Axford E., Dybdahl-Sissoko N., Oberste M.S., Wang T., Coyne C.B. (2011). The coxsackievirus B 3C protease cleaves MAVS and TRIF to attenuate host type I interferon and apoptotic signaling. PLoS Pathog..

[204] Rui Y., Su J., Wang H., Chang J., Wang S., Zheng W., Cai Y., Wei W., Gordy J.T., Markham R. (2017). Disruption of MDA5-Mediated Innate Immune Responses by the 3C Proteins of Coxsackievirus A16, Coxsackievirus A6, and Enterovirus D68. J. Virol..

[205] Qian S., Fan W., Liu T., Wu M., Zhang H., Cui X., Zhou Y., Hu J., Wei S., Chen H. (2017). Seneca Valley Virus Suppresses Host Type I Interferon Production by Targeting Adaptor Proteins MAVS, TRIF, and TANK for Cleavage. J. Virol..

[206] Xue Q., Liu H., Zhu Z., Yang F., Ma L., Cai X., Xue Q., Zheng H. (2018). Seneca Valley Virus 3C(pro) abrogates the IRF3- and IRF7-mediated innate immune response by degrading IRF3 and IRF7. Virology.

[207] Zhu X., Fang L., Wang D., Yang Y., Chen J., Ye X., Foda M.F., Xiao S. (2017). Porcine deltacoronavirus nsp5 inhibits interferon-β production through the cleavage of NEMO. Virology.

[208] Zhu X., Wang D., Zhou J., Pan T., Chen J., Yang Y., Lv M., Ye X., Peng G., Fang L. (2017). Porcine Deltacoronavirus nsp5 Antagonizes Type I Interferon Signaling by Cleaving STAT2. J. Virol..

[209] Zhao T., Yang L., Sun Q., Arguello M., Ballard D.W., Hiscott J., Lin R. (2007). The NEMO adaptor bridges the nuclear factor-kappaB and interferon regulatory factor signaling pathways. Nat. Immunol..

[210] St John S.E., Anson B.J., Mesecar A.D. (2016). X-Ray Structure and Inhibition of 3C-like Protease from Porcine Epidemic Diarrhea Virus. Sci. Rep..

[211] Ye G., Deng F., Shen Z., Luo R., Zhao L., Xiao S., Fu Z.F., Peng G. (2016). Structural basis for the dimerization and substrate recognition specificity of porcine epidemic diarrhea virus 3C-like protease. Virology.

[212] Yuan S. (2017). Studies on the molecular mechanism of porcine epidemic diarrhoea virus non-structural protein nsp7 inhibiting IFN-I signaling. Master’s Thesis.

[213] Romero-Brey I., Bartenschlager R. (2014). Membranous replication factories induced by plus-strand RNA viruses. Viruses.

[214] Den Boon J.A., Ahlquist P. (2010). Organelle-like membrane compartmentalization of positive-strand RNA virus replication factories. Annu. Rev. Microbiol..

[215] Kang H., Bhardwaj K., Li Y., Palaninathan S., Sacchettini J., Guarino L., Leibowitz J.L., Kao C.C. (2007). Biochemical and genetic analyses of murine hepatitis virus Nsp15 endoribonuclease. J. Virol..

[216] Ulferts R., Ziebuhr J. (2011). Nidovirus ribonucleases: Structures and functions in viral replication. RNA Biol..

[217] Deng X., Baker S.C. (2018). An “Old” protein with a new story: Coronavirus endoribonuclease is important for evading host antiviral defenses. Virology.

[218] Menachery V.D., Debbink K., Baric R.S. (2014). Coronavirus non-structural protein 16: Evasion, attenuation, and possible treatments. Virus Res..

[219] Egloff M.P., Benarroch D., Selisko B., Romette J.L., Canard B. (2002). An RNA cap (nucleoside-2′-O-)-methyltransferase in the flavivirus RNA polymerase NS5: Crystal structure and functional characterization. EMBO J..

[220] Becares M., Pascual-Iglesias A., Nogales A., Sola I., Enjuanes L., Zuniga S. (2016). Mutagenesis of Coronavirus nsp14 Reveals Its Potential Role in Modulation of the Innate Immune Response. J. Virol..

[221] Case J.B., Li Y., Elliott R., Lu X., Graepel K.W., Sexton N.R., Smith E.C., Weiss S.R., Denison M.R. (2018). Murine Hepatitis Virus nsp14 Exoribonuclease Activity Is Required for Resistance to Innate Immunity. J. Virol..

[222] Sur R., Lyte P.A., Southall M.D. (2008). Hsp27 regulates pro-inflammatory mediator release in keratinocytes by modulating NF-kappaB signaling. J. Investig. Dermatol..

[223] Vidyasagar A., Wilson N.A., Djamali A. (2012). Heat shock protein 27 (HSP27): Biomarker of disease and therapeutic target. Fibrogenesis Tissue Repair.

[224] Li Z., Chen F., Ye S., Guo X., Muhanmmad Memon A., Wu M., He Q. (2016). Comparative Proteome Analysis of Porcine Jejunum Tissues in Response to a Virulent Strain of Porcine Epidemic Diarrhea Virus and Its Attenuated Strain. Viruses.

[225] Zeng S., Zhang H., Ding Z., Luo R., An K., Liu L., Bi J., Chen H., Xiao S., Fang L. (2015). Proteome analysis of porcine epidemic diarrhea virus (PEDV)-infected Vero cells. Proteomics.

[226] Zhou Y.J., Zhu J.P., Zhou T., Cheng Q., Yu L.X., Wang Y.X., Yang S., Jiang Y.F., Tong W., Gao F. (2014). Identification of differentially expressed proteins in porcine alveolar macrophages infected with virulent/attenuated strains of porcine reproductive and respiratory syndrome virus. PLoS ONE.

[227] Fukagawa Y., Nishikawa J., Kuramitsu Y., Iwakiri D., Takada K., Imai S., Satake M., Okamoto T., Fujimoto M., Okita K. (2008). Epstein-Barr virus upregulates phosphorylated heat shock protein 27 kDa in carcinoma cells using the phosphoinositide 3-kinase/Akt pathway. Electrophoresis.

[228] Sun M., Yu Z., Ma J., Pan Z., Lu C., Yao H. (2017). Down-regulating heat shock protein 27 is involved in porcine epidemic diarrhea virus escaping from host antiviral mechanism. Vet. Microbiol..

[229] Mayer M.P. (2005). Recruitment of Hsp70 chaperones: A crucial part of viral survival strategies. Rev. Physiol. Biochem. Pharm..

[230] Chen H., Ning X., Jiang Z. (2017). Caspases control antiviral innate immunity. Cell Mol. Immunol..

[231] Kim Y., Lee C. (2014). Porcine epidemic diarrhea virus induces caspase-independent apoptosis through activation of mitochondrial apoptosis-inducing factor. Virology.

[232] Kumar S. (2007). Caspase function in programmed cell death. Cell Death Differ..

[233] Ron D., Walter P. (2007). Signal integration in the endoplasmic reticulum unfolded protein response. Nat. Rev. Mol. Cell. Biol..

[234] Fung T.S., Liu D.X. (2014). Coronavirus infection, ER stress, apoptosis and innate immunity. Front. Microbiol..

[235] Fung T.S., Liao Y., Liu D.X. (2016). Regulation of Stress Responses and Translational Control by Coronavirus. Viruses.

[236] Zou D., Xu J., Duan X., Xu X., Li P., Cheng L., Zheng L., Li X., Zhang Y., Wang X. (2019). Porcine epidemic diarrhea virus ORF3 protein causes endoplasmic reticulum stress to facilitate autophagy. Vet. Microbiol..

